# Bioinformatic analysis of the *LCN2–SLC22A17–MMP9* network in cancer: The role of DNA methylation in the modulation of tumor microenvironment

**DOI:** 10.3389/fcell.2022.945586

**Published:** 2022-09-21

**Authors:** Saverio Candido, Barbara Tomasello, Alessandro Lavoro, Luca Falzone, Giuseppe Gattuso, Angela Russo, Sabrina Paratore, James A. McCubrey, Massimo Libra

**Affiliations:** ^1^ Department of Biomedical and Biotechnological Sciences, University of Catania, Catania, Italy; ^2^ Research Center for Prevention, Diagnosis and Treatment of Cancer, University of Catania, Catania, Italy; ^3^ Department of Drug and Health Sciences, University of Catania, Catania, Italy; ^4^ Epidemiology Unit, IRCCS Istituto Nazionale Tumori “Fondazione G. Pascale”, Naples, Italy; ^5^ Pathological Anatomy Unit, ARNAS Garibaldi Hospital, Catania, Italy; ^6^ Department of Microbiology and Immunology, Brody School of Medicine, East Carolina University, Greenville, NC, United States

**Keywords:** LCN2, SLC22A17, MMP9, cancer, DNA methylation, gene expression, bioinformatic, ferroptosis

## Abstract

Several features of cancer cells such as proliferation, invasion, metastatic spreading, and drug resistance are affected by their interaction with several tumor microenvironment (TME) components, including neutrophil gelatinase-associated lipocalin (NGAL), solute carrier family 22 member 17 (SLC22A17), and matrix metallopeptidase 9 (MMP9). These molecules play a key role in tumor growth, invasion, and iron-dependent metabolism of cancer cells. However, the precise epigenetic mechanisms underlying the gene regulation of *Lipocalin 2* (*LCN2*), *SLC22A17*, and *MMP9* in cancer still remain unclear. To this purpose, computational analysis was performed on TCGA and GTEx datasets to evaluate the expression and DNA methylation status of *LCN2*, *SLC22A17*, and *MMP9* genes in different tumor types. Correlation analysis between gene/isoforms expression and DNA methylation levels of *LCN2*, *SLC22A17*, and *MMP9* was performed to investigate the role of DNA methylation in the modulation of these genes. Protein network analysis was carried out using reverse phase protein arrays (RPPA) data to identify protein–protein interactions of the *LCN2*–*SLC22A17*–*MMP9* network. Furthermore, survival analysis was performed according to gene expression and DNA methylation levels. Our results demonstrated that *LCN2* and *MMP9* were mainly upregulated in most tumor types, whereas *SLC22A17* was largely downregulated, representing a specific hallmark signature for all gastrointestinal tumors. Notably, the expression of *LCN2*, *SLC22A17*, and *MMP9* genes was negatively affected by promoter methylation. Conversely, intragenic hypermethylation was associated with the overexpression of *SLC22A17* and *MMP9* genes. Protein network analysis highlighted the role of the *LCN2*–*SLC22A17*–*MMP9* network in TME by the interaction with fibronectin 1 and claudin 7, especially in rectal tumors. Moreover, the impact of expression and methylation status of *LCN2*, *SLC22A17*, and *MMP9* on overall survival and progression free interval was tumor type–dependent. Overall, our analyses provide a detailed overview of the expression and methylation status of *LCN2*, *SLC22A17*, and *MMP9* in all TCGA tumors, indicating that the *LCN2–SLC22A17–MMP9* network was strictly regulated by DNA methylation within TME. Our findings pave the way for the identification of novel DNA methylation hotspots with diagnostic and prognostic values and suitable for epi-drug targeting.

## Introduction

During tumor development, the cancer cells are involved in intricate and dynamic interactions with the surrounding extracellular matrix (ECM) and with different cell types, including fibroblasts, endothelial cells, and immune cells. Cell–cell interaction and several signaling molecules ensure cell communication within the tumor microenvironment (TME) (Whiteside, 2008). In the last decade, numerous studies have highlighted the role of TME in several cancer features, including survival, invasion, metastasis, immune escape, and drug resistance. In this context, the *LCN2–SLC22A17–MMP9* (lipocalin 2—solute carrier family 22 member 17—matrix metallopeptidase 9) pathway contributes to the establishment and remodeling of TME, which is responsible for the tumor progression (Kessenbrock et al., 2010; Crescenzi et al., 2021). For instance, the MMP9–NGAL complex is involved in the ECM degradation and chevage of some adhesion proteins, thus, promoting cell migration and invasion (Candido et al., 2016a; Huang, 2018). Furthermore, MMP9 suppresses anti-tumor immunity modifying several ligands and receptors of the immune signature pathways, including T-cell chemoattractant CXC receptor 3 ligands (Juric et al., 2018). NGAL is also involved in iron homeostasis, mediating either the uptake of extracellular iron or the removal of intracellular iron with different effects on tumor cells depending on the TME context. The iron trafficking is mediated by the interaction of NGAL with its receptor resulting in NGAL–SLC22A17 complex internalization by receptor-mediated endocytosis, whereas NGAL–SLC22A17 complex recycling is involved in the iron efflux. These mechanisms are affected by specific isoforms of the *SLC22A17* gene. In particular, it has been reported that both *SLC22A17* isoforms 1 and 2 are bilateral iron transporters, while *SLC22A17* isoform 3 could exclusively mediate iron influx in esophageal cancer cells (Candido et al., 2014; Xiao et al., 2017).

The increase of intracellular iron results in iron-dependent growth pathways activation leading to survival and apoptosis inhibition. Conversely, the intracellular iron depletion leads to Hypoxia-inducible factor 1 *α* (HIF-1α) activation and Bcl-2 interacting mediator of cell death (BIM)–dependent apoptosis induction (Bolignano et al., 2010). Recently, the role of NGAL–SLC22A17 interaction in ferroptosis has been investigated in colorectal cancer, suggesting that NGAL contributes to prevent iron-dependent oxidative stress damage by decreasing the iron intracellular concentration of tumor cells (Chaudhary et al., 2021). This evidence is supported by the results demonstrating that the targeting of NGAL or SLC22A17 sensitizes to ferroptosis inducers through the intracellular iron accumulation in liver and pancreatic cancer (Yao et al., 2021; Liu et al., 2021). However, cell uptake of either apo-NGAL (iron-free form) or holo-NGAL (iron-loaded form) had different effects on iron-dependent ferroptosis, highlighting the need for further studies to better understand the precise role of NGAL-mediated iron trafficking (Meier et al., 2021).

Despite the role of NGAL and MMP9 in tumor invasion and metastasis has been deeply investigated, the exact involvement of SLC22A17 in cancer development has not been yet clarified. In particular, further investigations should be carried out to better clarify the relationship between SLC22A17 and iron metabolism, whose imbalance either promotes tumor growth or induces oxidative stress–mediated ferroptosis. In this context, the genetic and epigenetic factors affecting the expression of *LCN2*, *SLC22A17*, and *MMP9* need to be investigated to evaluate their diagnostic and prognostic values and their use as potential pharmacological targets.

As widely reported in the literature, these proteins interact at different levels; for instance, SCL22A17 is the receptor for LCN2, the latter increases the activity of MMP9, and both genes are transcriptionally regulated by the same factors, including NF-kB (Yang et al., 2019; Kim et al., 2022). This evidence suggests that their interaction plays a key role in several cellular processes, including the perturbation of the TME (Candido et al., 2014).

To date, a comprehensive view of the functioning and regulation of this network in cancer is lacking. Therefore, our study represents an overview which aims to investigate the genetic and epigenetic regulation of the *LCN2*–*SLC22A17*–*MMP9* network. On this basis, we conducted an *in silico* study to evaluate the role of *LCN2*, *SLC22A17*, and *MMP9* genes and related isoforms in different tumor types by following specific workflow steps. First, we analyzed both the expression and methylation data available on TCGA and GTEx datasets. Second, correlation analyses between the methylation status of CG hotspots and the expression levels of these genes were carried out, showing that methylation affects their expression in most tumor types. Subsequently, correlation analysis between the expression of *LCN2*, *SLC22A17*, and *MMP9* genes and RPPA protein levels was performed to identify their involvement in cellular processes and cancer pathways. Finally, the prognostic and diagnostic values were evaluated for each gene and relative DNA methylation hotspots.

Overall, this study focused on the role of DNA methylation in the gene regulation of *LCN2*, *SLC22A17*, and *MMP9* and their interaction with several proteins, including those involved in the TME, providing a complete and accurate source data which is useful as a starting point for future advances in the field of epigenetic regulation of TME.

## Materials and methods

### Data collection from public repositories TCGA and GTEx

UCSC Xena Functional Genomics Explorer (https://xenabrowser.net/, accessed on 13th January 2022) was used to retrieve the expression levels of *LCN2*, *SLC22A17*, and *MMP9* genes and related isoforms from the TCGA TARGET GTEx cohort (*n* = 9,807 TCGA tumor and *n* = 7,414 GTEx normal samples), selecting the gene expression RNAseq (*n* = 19,131) and transcript expression RNAseq (*n* = 19,129) datasets. This cohort allows comparing the expression data relative to the tumor (TCGA) and normal (GTEx) samples since they were re-analyzed according to UCSC RNA-seq Compendium to obtain data homogenization. Expression data were indicated as log2 (fpkm + 0.001) transcript expression unit.

### Datasets for expression, methylation, and protein analyses in TCGA tumor samples

Gene/isoforms expression and DNA methylation levels of *LCN2*, *SLC22A17*, and *MMP9* were obtained from Xena Explorer (accessed on 13th January 2022), analyzing the TCGA Pan-Cancer cohorts. Gene expression RNAseq (*n* = 10,535) and transcript expression RNAseq (*n* = 10,534) datasets were selected to retrieve log2 (fpkm + 0.001) expression values. DNA methylation (Methylation 450K) (*n* = 9,639) dataset was interrogated using the Xena UCSC tool to download the methylation values (beta value) of CG probesets relative to *LCN2*, *SLC22A17*, and *MMP9* genes. All available protein levels for each TCGA tumor type were retrieved directly from the protein expression—RPPA (*n* = 7,744) dataset.

### Survival analysis

UCSC Xena tool (accessed on 18th February 2022) was used to retrieve the data of the overall survival (OS) and progression free interval (PFI) in TCGA Pan-Cancer cohorts. The Log-rank-test and p-value were achieved, and prognostic significance was manually annotated.

### Statistical analyses and data visualization

R software version 4.1.0 was used to perform differential and correlation analyses. To evaluate the difference between the comparison groups, the fold change (FC) was calculated using the formula:

$$\pm 2^{\left|\text{mean}\left(\text{Group}2\;\text{values}\right)-\text{mean}\left(\text{Group}1\;\text{values}\right)\right|}.$$

The mean of each group was calculated using log2 values. The FC sign was positive if the mean of group2 was higher than that of group1; conversely, it was negative when the mean1 was higher than mean2. The statistical significance between the comparison groups was evaluated using a two-tailed unpaired *t*-test using the t. test function included in the Rcmdr package. Pearson’s correlation test was performed using the cor_test function of the rstatix package. Box plot shaped using the ggplot function counted in the ggplot2 package.

Heatmap and clustering analyses were performed using Heatmapper (http://heatmapper.ca) (accessed on 10th February 2022) according to the developers’ instructions. Volcano plot analyses were carried out using the publicly available tool VolcaNoseR (https://huygens.science.uva.nl/VolcaNoseR2/) (accessed on 22nd February 2022). Network representations of gene–protein and protein–protein correlations were generated using Cytoscape 3.8.2 (https://cytoscape.org/) (accessed on 24th February 2022). String tool (https://string-db.org/) (accessed on 25th February 2021) was used to retrieve and visualize protein–protein interaction networks and functional enrichment analysis.

## Results

### Gene expression profiling of *LCN2*, *SLC22A17*, and *MMP9* in tumor and normal samples

**FIGURE 1:**
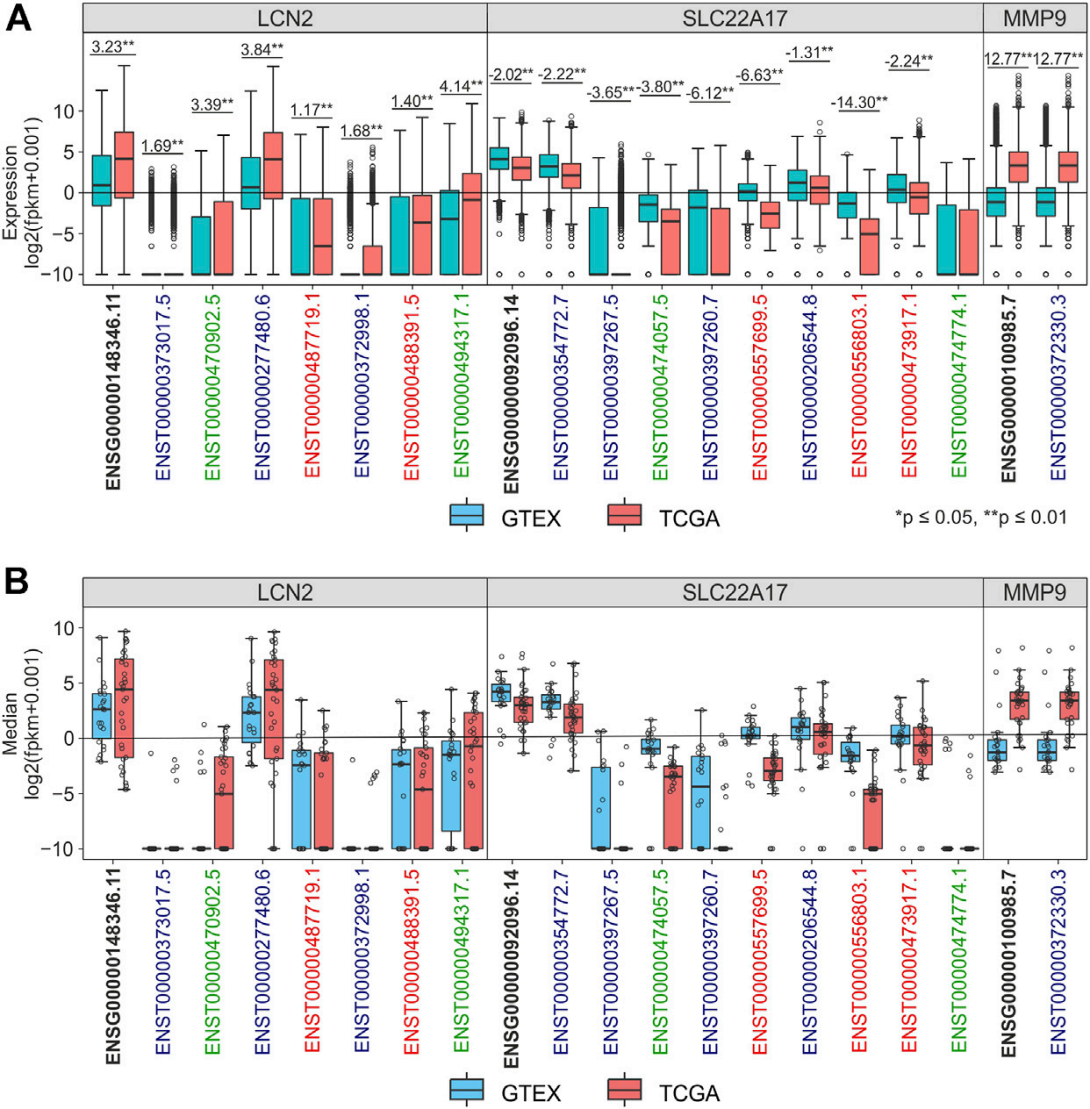
Differential analysis of *LCN2*, *SLC22A17*, and *MMP9* expression levels in TCGA tumor and GTEx normal samples. **(A)** Expression levels of *LCN2*, *SLC22A17*, and *MMP9* genes and isoforms in normal (blue) and tumor samples (red). Fold Change was reported for each comparing group. **(B)** Distribution of expression median values calculated for the gene and isoforms (empty circles) is shown for each tumor type and matched normal tissues. Black bold labels indicate the *LCN2*, *SLC22A17*, and *MMP9* gene expression (ENSG), including all spliced transcripts (ENST). Coding isoforms: blue; non-coding isoforms: green; retained intron isoforms: red.

The role in tumorigenesis of *LCN2*, *SLC22A17*, and *MMP9* genes and their gene isoforms as key players of TME interaction was assessed by performing differential transcriptomic analysis of these targets between TCGA tumor samples and GTEx normal tissues. In particular, the expression of gene (ENSG) and spliced transcripts (ENST) was individually evaluated for each gene using RNA-seq data from the TCGA TARGET GTEx cohort (Figure 1; Supplementary Figure S1). The results indicated that the expression of the *LCN2* gene (ENSG00000148346.11) and all its isoforms was upregulated in tumor samples compared to normal tissues. Notably, the ENST00000277480.6 isoform showed a conspicuous increment of 3.84 FC (*p* ≤ 0.01), while the other *LCN2* isoforms were found to be weakly expressed in both normal and tumor samples (Figure 1A; Supplementary Table S2). A similar trend was observed considering the median value of each gene/isoform computed for each tumor type (Figure 1B; Supplementary Table S2). On the other hand, the expression levels of the *SLC22A17* gene and its isoforms were downregulated in tumor samples compared to normal tissues by analyzing collectively all tumor samples (Figure 1A) or by tumor type (Figure 1B). Among the *SLC22A17* isoforms, the coding isoforms ENST00000354772.7 and ENST00000206544.8 showed the highest expression levels in both tumor and normal samples (Figure 1A). As regards the *MMP9* gene, only ENST00000372330.3 isoform was available for all tumors and normal samples, and its expression values were identical to those of the ENSG00000100985.7 gene. Overall, the expression levels of *MMP9* were upregulated in tumors compared to normal tissues (Figures 1A and B; Supplementary Table S2).

**FIGURE 2:**
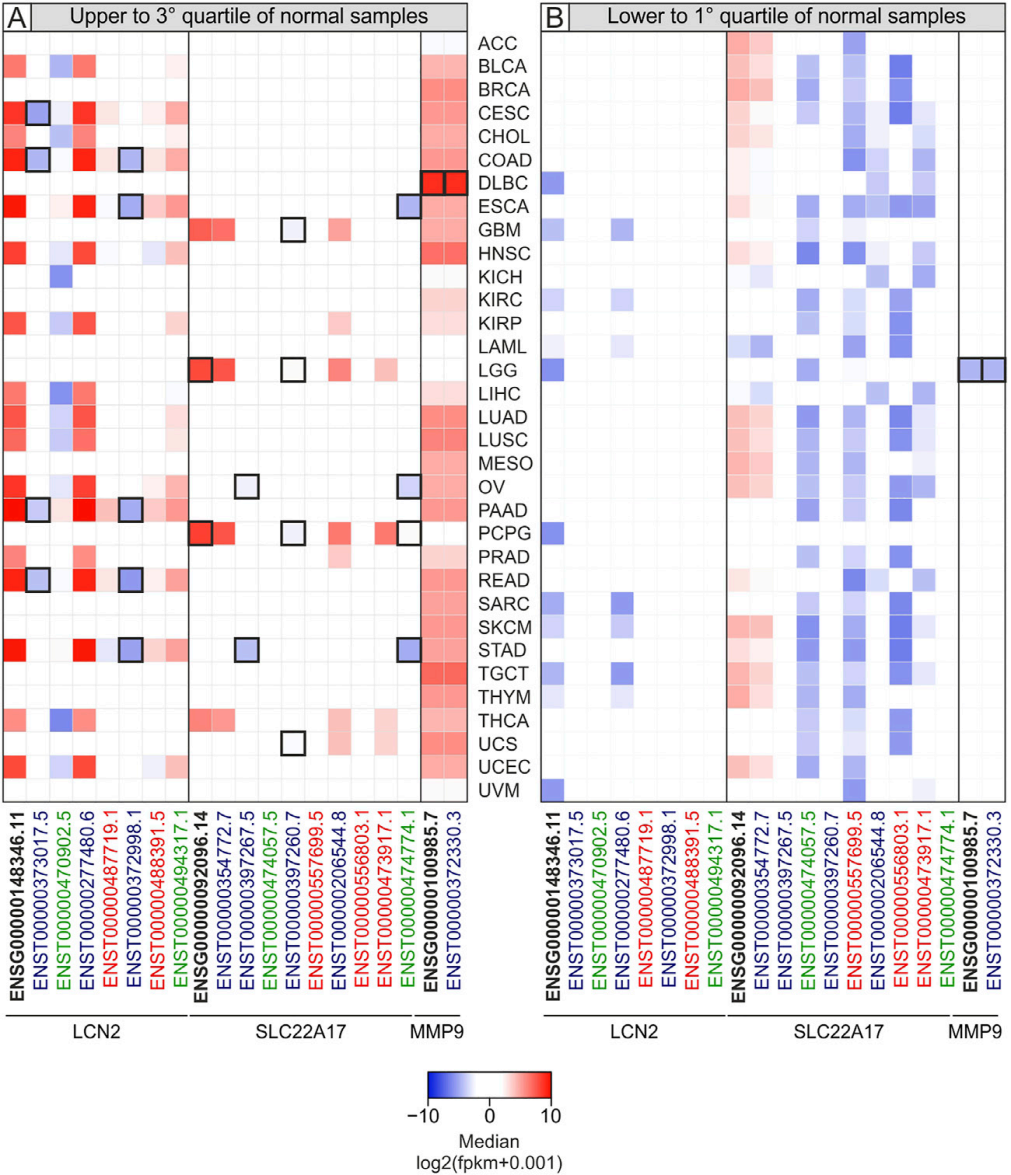
Heatmap of median expression levels for *LCN2*, *SLC22A17*, and *MMP9* genes and isoforms in tumor samples according to interquartile range of normal samples median values. The median values of each tumor type higher than the 3rd quartile **(A)** or lower than the 1st quartile of normal tissues **(B)** are reported. The negative median values (lower expression) are shown as blue squares, while positive ones (higher expression) are indicated in red. Black border indicates the median values of each tumor type that are also outliers. Black bold labels indicate the *LCN2*, *SLC22A17*, and *MMP9* genes (ENSG). Coding ENST isoforms: blue; non-coding ENST isoforms: green; retained intron ENST isoforms: red.

To discover the *LCN2*, *SLC22A17*, and *MMP9* genes and isoforms that were highly up- or down-regulated in tumors, the median expression value of each gene/isoform for each tumor type was compared with the interquartile range (IQRn) of the corresponding normal tissues. This analysis allowed us to identify the median levels of *LCN2*, *SLC22A17*, and *MMP9* genes and isoforms of each tumor type that were upper to 3rd or lower to 1st quartiles of corresponding normal tissue (Figure 2; Supplementary Table S2). Furthermore, an outlier analysis was performed to identify the median value of each gene/isoform associated with a specific tumor type that was abnormally up- or down-regulated with respect to the overall median value (Figure 2, Black border). The analysis revealed that the expression of both the *LCN2* gene (ENSG00000148346.11) and ENST00000277480.6 isoform was upper than the 3rd quartile of normal samples in 51.5% of all TCGA tumors (*N* = 33), including PAAD, STAD, ESCA, READ, and COAD. Notably, all the median log_2_ fpkm values of both gene and isoform were higher than 0 in all tumors (Figure 2A, red square). In addition, the ENSG00000148346.11 median values were lower than the 1st quartile in 33.3% of tumors, including LGG, DLBC, PCPG, and UVM (Figure 2B, blue square). When the analysis was performed for the *SLC22A17* gene, its median values were higher than the 3^rd^ quartile of normal values in LGG, GBM, PCPG, and THCA and for ENST00000354772.7 and ENST00000206544.8 isoforms. Notably, the median values of ENSG00000092096.14 were outliers for LGG and PCPG (Figure 2A, thick black border). Interestingly, the median values of the *SLC22A17* gene and three isoforms (ENST00000354772.7, ENST00000474057.5, and ENST00000557699.5) were found to be lower than the 1st quartile of normal values in more than 60% of tumors (Figure 2B; Supplementary Table S2).

As regards *MMP9*, it was observed that the median values of gene expression were upper than the 3rd quartile of normal values in 30 tumors (90.9%) except for LAML, LGG, and PCPG (Figure 2A; Supplementary Table S2). Of these, only LGG showed *MMP9* median level markedly lower than the 1st quartile of normal values, such that it was considered a lower outlier (Figure 2B).

**FIGURE 3:**
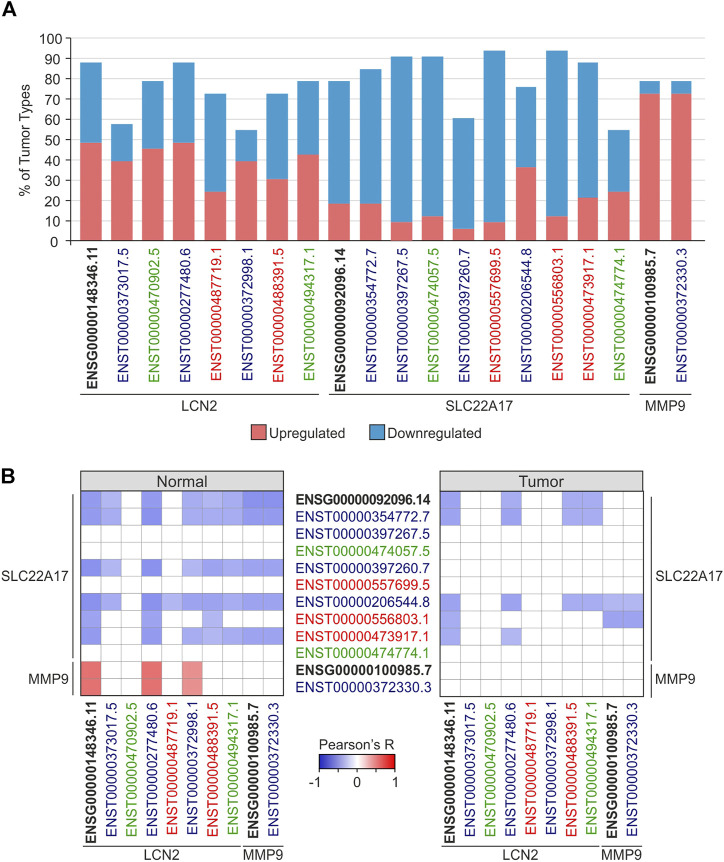
Expression and correlation analyses of gene (ENSG) and splicing isoforms (ENST) of LCN2, SLC22A17, and MMP9 in normal and tumoral tissues. **(A)** Percentage of tumor types in which *LCN2*, *SLC22A17*, and *MMP9* expression levels were up (red) or downregulated (blue) compared to normal tissues. Only tumors showing FC ≥ 1.4 or ≤ −1.4 (*p* ≤ 0.05) are included in the analysis. **(B)** Correlation analysis between *LCN2*, *SLC22A17*, and *MMP9* genes and isoforms expression in normal and tumor samples. Tumor and normal samples were analyzed collectively. Correlation with Pearson’s r ≥ 0.3 or ≤ −0.3 and *p* ≤ 0.05 were included in the heatmap. Black bold labels indicate the *LCN2*, *SLC22A17*, and *MMP9* genes (ENSG). Coding ENST isoforms: blue; non-coding ENST isoforms: green; retained intron ENST isoforms: red.

To deeply investigate the modulation of *LCN2*, *SC22A17*, and *MMP9* network in tumors, differential analysis was performed by comparing each tumor type with the relative normal tissue (Supplementary Tables S1, S3). The analysis revealed that the *LCN2* gene and ENST isoforms were markedly differentially expressed (FC ≥ 1.4 or ≤ −1.4; *p* ≤ 0.05) in nearly 60% of tumors except for the coding isoforms ENST00000373017.5 (30.3%) and ENST00000372998.1 (33.3%). Notably, *LCN2* expression levels were upregulated in about half of the selected tumors (Figure 3A; Supplementary Table S3). Unlike *LCN2*, *SLC22A17* was predominantly downregulated in more than 75% of tumor types, showing statistical significance (FC ≥ 1.4 or ≤ −1.4; *p* ≤ 0.05) for the gene and seven of its isoforms. Interestingly, the expression levels of *MMP9* were upregulated in 28 tumor types and downregulated only in LAML and THYM, whereas no statistical difference was achieved in DLBC, MESO, and UVM (Figure 3A; Supplementary Table S3).

Finally, the correlation analysis between genes and isoforms of *LCN2*, *SLC22A17*, and *MMP9* in both normal and tumor samples was performed to detect any interaction between the three genes, including the relative isoforms (Figure 3; Supplementary Table S4). The results in normal tissues showed that *LCN2* isoforms, except for the non-coding ENST00000470902.5 isoform, were negatively correlated (Pearson’s r ≤ −0.3; *p* ≤ 0.05) with the *SLC22A17* gene and five related isoforms, including three of four coding isoforms. Conversely, *LCN2* gene and ENST00000277480.6 and ENST00000372998.1 coding isoforms were positively correlated (Pearson’s r ≥ 0.3; *p* ≤ 0.05) with *MMP9* expression levels. However, *MMP9* was negatively correlated with the *SLC22A17* gene and four related isoforms (Figure 3B left panel; Supplementary Table S4). When correlation analysis was carried out in tumor samples, *SLC22A17* was negatively correlated with both *LCN2* and *MMP9*, showing few correlation pairs compared to those obtained for normal samples (Figure 3B right panel; Supplementary Table S4).

Overall, differential analysis revealed that *LCN2* and *MMP9* were widely upregulated in several tumor types, while an opposite trend was observed for *SLC22A17*, suggesting its potential role in tumor suppression. In addition, no difference was observed comparing the expression profile of *LCN2*, *SC22A17*, and *MMP9* genes and relative isoforms, indicating that, for these genes, alternative splicing plays a marginal role in the modulation of their network. Performing correlation analysis among the three genes and their isoforms allowed us to observe how the interactions of genes/isoforms change in tumor samples compared to normal tissues. Specifically, the observed reduction in correlation pairs demonstrated how they are differently associated in tumors, pointing out that this network results in dysregulation in cancer.

### Correlation and pathway analyses of *LCN2*, *SLC22A17*, and *MMP9* expression and RPPA protein levels

**FIGURE 4:**
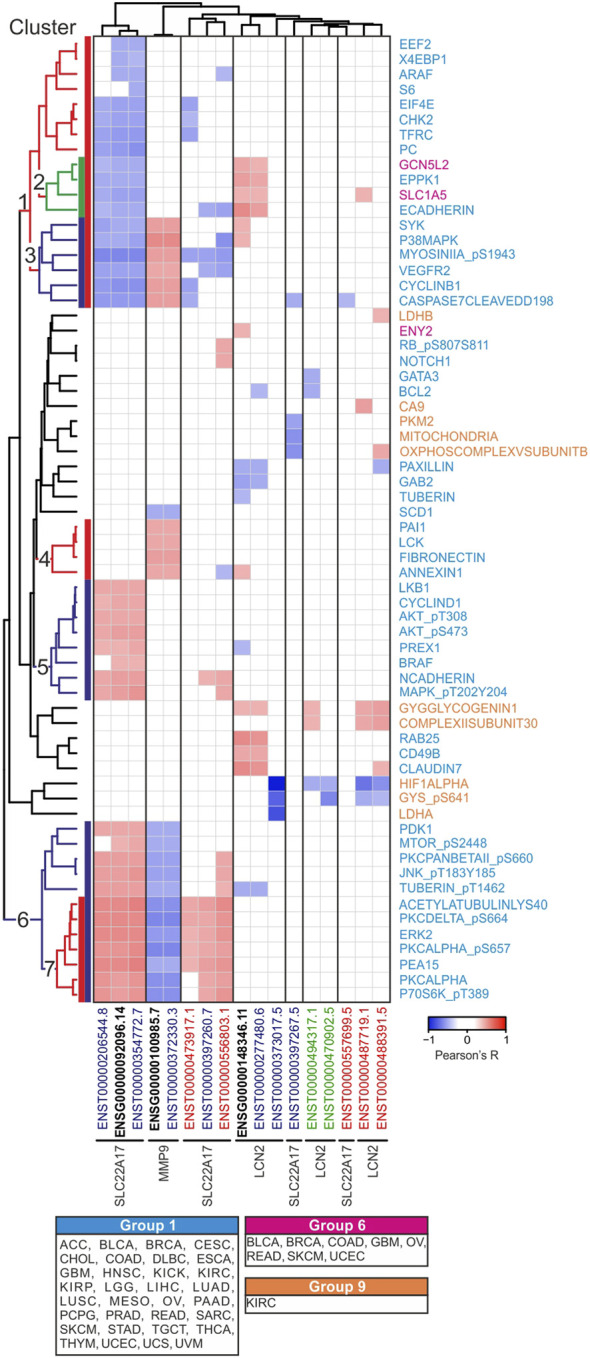
Correlation analysis between *LCN2*, *SLC22A17*, and *MMP9* gene/isoforms expression and protein levels in PANCAN tumor samples. Correlation with Pearson’s r ≥ 0.3 or ≤ −0.3 and *p* ≤ 0.05 are included in the heatmap. The average linkage clustering method and Euclidean distance measurement method were applied for grouping similar data in seven representative clusters. Each protein is labeled with three different colors corresponding to 1, 6, and 9 tumor groups (Supplementary Table S6). Black bold labels indicate the *LCN2*, *SLC22A17*, and *MMP9* genes (ENSG). Coding ENST isoforms: blue; non-coding ENST isoforms: green; retained intron ENST isoforms: red.

To depict relations among *LCN2*, *SLC22A17*, and *MMP9* and to analyze the structures of network that arise from these relations and the RPPA proteins, different approaches were applied. First, correlation analysis was performed between *LCN2*, *SLC22A17*, and *MMP9* gene/isoforms expression and protein levels downloaded from the RPPA dataset included in the TCGA PAN-CANCER cohort (Figure 4; Supplementary Table S5) to define the protein network interaction of these three genes. Since the expression levels of proteins were not available for all tumor types, the proteins were divided into nine different “tumor-groups” based on tumor types, where they were analyzed (Supplementary Table S6). Significantly correlated gene/isoform–protein pairs were sorted into seven major clusters using the average linkage clustering method (Figure 4; Supplementary Table S7). Furthermore, gene ontology analysis (STRING) was performed for each protein cluster to assess the matching of proteins with the KEGG pathways (Supplementary Table S7) to identify the role of *LCN2*, *SLC22A17*, and *MMP9* genes in cellular processes and cancer pathways.

The analyses revealed that the *LCN2* gene and its isoform ENST00000277480.6 showed a positive correlation only with protein cluster 2 comprising EPPK1 and ECADHERIN (tumor group 1) and SLC1A5 and GCN5L2 (tumor group 6). However, no significant pathway enrichment was achieved (Figure 4).

As regards *SLC22A17*, the gene and related isoforms ENST00000206544.8 and ENST00000354772.7 were negatively correlated (Pearson’s r ≤ −0.3; *p* ≤ 0.05) with some proteins of cluster 1, whereas they showed a positive correlation (Pearson’s r ≥ 0.3; *p* ≤ 0.05) with proteins of both cluster 5 and cluster 6. The protein–protein interaction analysis of these clusters using the STRING tool showed many “functional protein associations” with high enrichment strength (>2), which were involved in several signaling pathways related to cancer, such as EGFR tyrosine kinase inhibitor resistance, VEGF and FoxO signaling pathways, and ErbB signaling pathway (Supplementary Table S7).

Finally, *MMP9* expression was found to be correlated with three different protein clusters (Figure 4). Specifically, *MMP9* was negatively correlated with proteins of cluster 6, which were mainly involved in the tyrosine kinase transduction pathway, as previously reported. Conversely, a positive correlation was observed for both clusters 3 and 4. Of these, only the proteins in cluster 3 matched the VEGF signaling pathway, while no significant pathway enrichment was observed for cluster 4 (Supplementary Table S7).

### Correlation and network analyses of *LCN2*, *SLC22A17*, and *MMP9* expression and RPPA protein levels by tumor type

**FIGURE 5:**
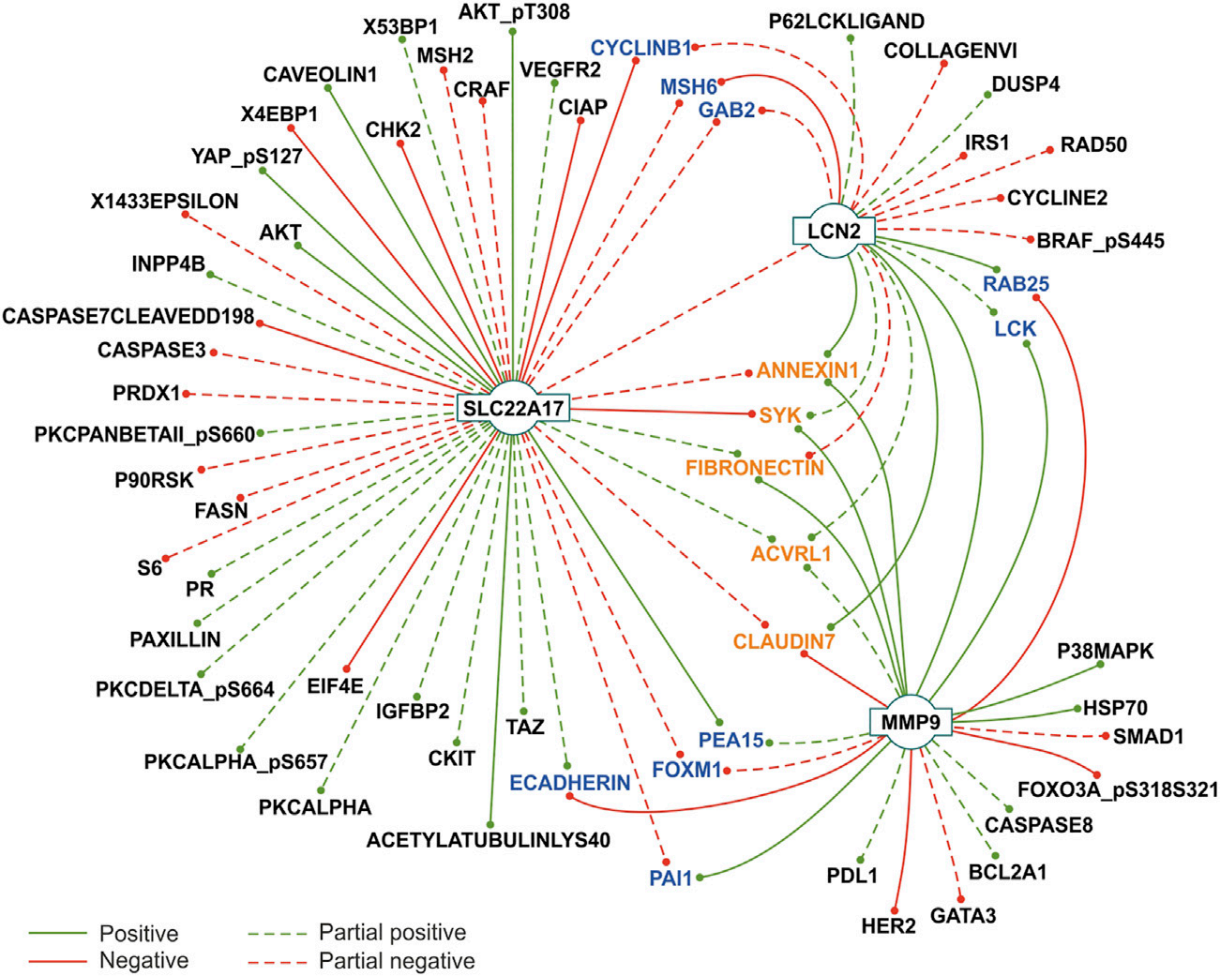
Correlation analysis between *LCN2*, *SLC22A17*, and *MMP9* gene expression and protein levels in tumor types. Protein-gene interaction showing correlation (Pearson’s r ≥ 0.3 or ≤ −0.3; *p* ≤ 0.05) in at least five tumor types are reported. The concordance among Pearson’s values was defined as the degree of agreement among the correlation sign. Partial concordance: dotted interaction lines. Total concordance: solid interaction lines. Proteins interacting with two genes are represented in blue, while proteins interacting with all three genes are represented in orange. The network was obtained from Cytoscape 3.8.2 (https://cytoscape.org/).

To deeply investigate the interaction between *LCN2*, *SLC22A17*, and *MMP9* genes and RPPA proteins in cancer, correlation analysis was performed according to the tumor type (Supplementary Table S8). Moreover, to identify proteins significantly correlated with the expression of these genes, Cytoscape network analysis was performed by including only gene–proteins pairs that were significantly correlated (*r* ≥ 0.3 or ≤ −0.3; *p* ≤ 0.05) in at least five tumor types (Figure 5).

This analysis confirmed the association between the main factors of the network; in fact, *LCN2* expression was positively correlated with *MMP9* (full concordance in eight tumor types), while partial concordance was observed with *SLC22A17*, which showed negative correlation in seven tumors and a positive one in three tumor types (Figure 5; Supplementary Table S8). Interestingly, the *SLC22A17* gene showed more protein-interactions than *MMP9* and *LCN2*. In particular, the expression of *SLC22A17* was correlated with 14 proteins with complete concordance. Specifically, seven proteins (e.g., AKT and CAVEOLIN1) were positively correlated, and the others (e.g., CHK2 and CIAP) displayed a negative correlation (Figure 5; Supplementary Table S8). Furthermore, a total concordance was observed between *MMP9* expression and 12 proteins. Specifically, seven proteins showed a positive correlation, for instance, FIBRONECTIN (tumors *n* = 12), PAI1 (tumors *n* = 11), and LCK (tumors *n* = 10), while five proteins (e.g., ECADHERIN and CLAUDIN7) were negatively correlated with *MMP9* expression (Figure 5; Supplementary Table S8). Finally, *LCN2* expression was positively correlated with ANNEXIN1, RAB25, and CLAUDIN7 with complete correlation concordance (solid lines), while only MSH6 showed a negative interaction with *LCN2* (Figure 5).

It was also observed that some proteins concomitantly correlated with two genes of the LCN2–SLC22A17–MMP9 network (Figure 5, blue labeled), while ANNEXIN1, SYK, FIBRONECTIN, ACVRL1, and CLAUDIN7 were simultaneously correlated with all three genes.

**FIGURE 6:**
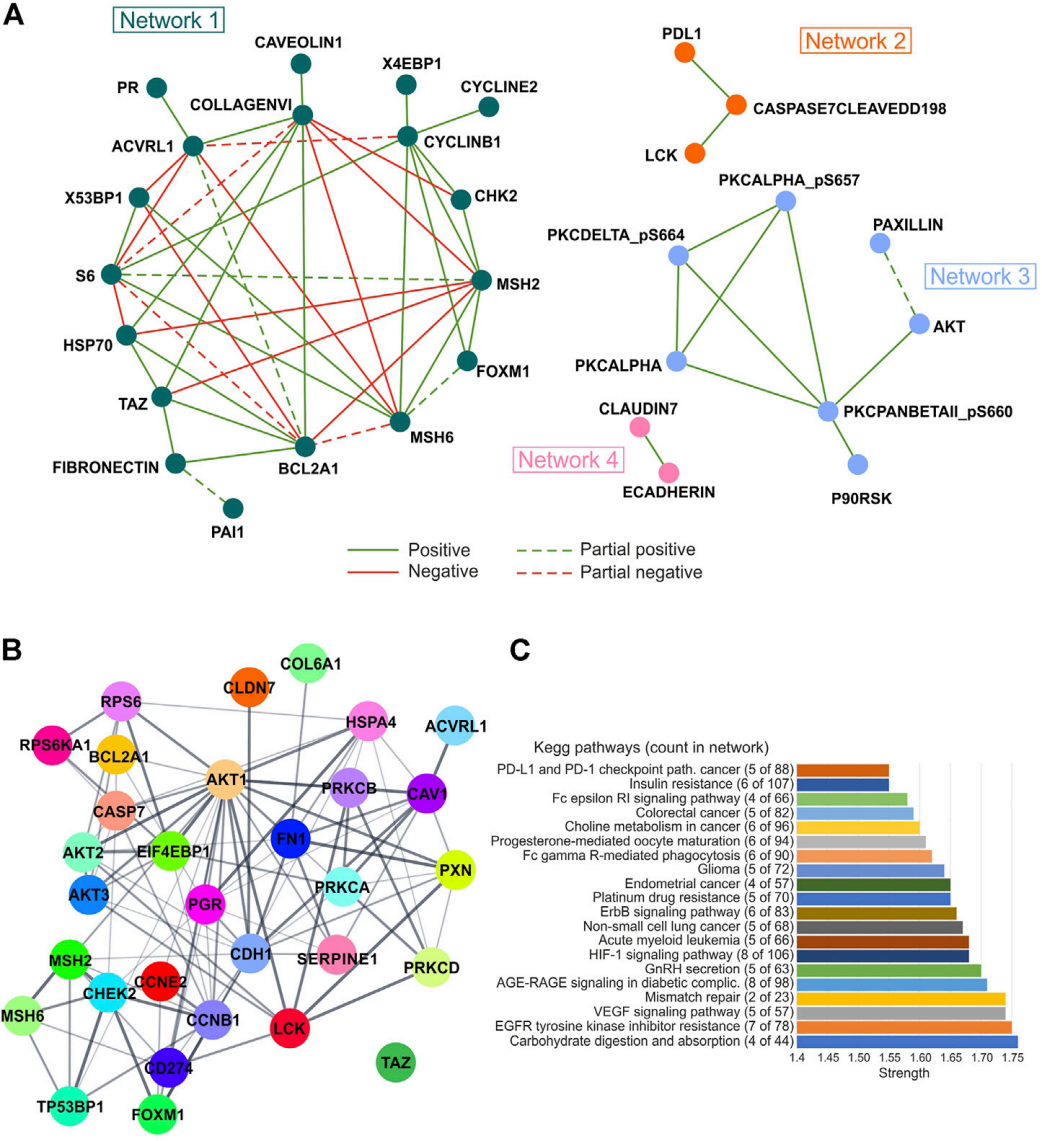
Correlation and pathway analysis of protein levels analyzed in each tumor type. **(A)** Protein–protein interaction showing correlation (Pearson’s r ≥ 0.3 or ≤ −0.3; *p* ≤ 0.05) in at least 20 tumor types are represented. The concordance among Pearson’s value means was defined as a degree of agreement among the correlation sign. Partial concordance: dotted interaction lines. Total concordance: solid interaction lines. The network was obtained from Cytoscape 3.8.2 (https://cytoscape.org/). **(B)** STRING network analysis (https://string-db.org/) of selected proteins significantly correlated with each other (Pearson’s r ≥ 0.3 or ≤ −0.3; *p* ≤ 0.05). Edge thickness indicates the strength of data support. **(C)** Gene ontology analysis according to STRING algorithms. Strength of functional enrichments and count of proteins in each KEGG pathway are indicated.

To identify the most significant protein–protein interactions, network analysis was also performed on significant correlation pairs (*r* ≥ 0.3 or ≤ −0.3; *p* ≤ 0.05) in at least 20 tumor types, thus, obtaining a total of four networks. Of note, networks 2, 3, and 4 were only characterized by positive interactions, whereas both positive and negative interactions were observed for network 1 with different degrees of concordance (Figure 6A; Supplementary Table S8).

The STRING network analysis performed on the proteins of the four networks revealed a high number of protein interactions. Interestingly, AKT1 was the node with the most interactions, while no interaction was observed for TAZ protein (Figure 6B). Furthermore, carbohydrate digestion and absorption, EGFR tyrosine kinase inhibitor resistance, VEGF signaling pathway, and mismatch repair were the most enriched KEGG pathways (strength >1.74) by analyzing all correlated proteins using the STRING tool (Figure 6C).

### OS and PFI analyses of *LCN2*, *SLC22A17*, and *MMP9* expression in TCGA PAN-CANCER samples

**FIGURE 7:**
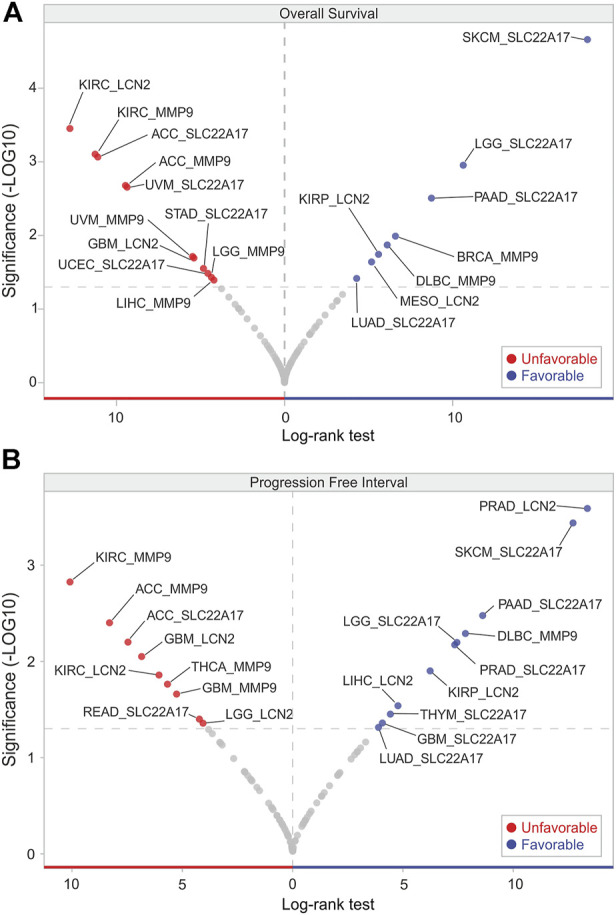
OS and PFI analyses according to *LCN2*, *SCL22A17*, and *MMP9* gene expression. Volcano plot visualization of tumor types in which *LCN2*, *SCL22A17*, and *MMP9* gene expression affected the patient’s overall survival **(A)** and progression free interval **(B)**. The genes showing significant log-rank test (*p* ≤ 0.05) are represented in red (unfavorable) or in blue (favorable) according to their prognostic significance in each tumor type.

To assess whether the expression of *LCN2*, *SLC22A17*, and *MMP9* genes was predictive of the survival outcome in cancer patients, the UCSC Xena tool was used to obtain the OS and PFI data according to their gene expression in all tumor types included in the TCGA PAN-CANCER cohort (Supplementary Table S9). Volcano plot analysis of OS revealed that *MMP9* was an unfavorable prognostic factor in five tumor types (ACC, KIRC, LGG, LIHC, and UVM), while it was favorable only in BRCA and DLBC (Figure 7A). In contrast, based on prognostic significance, an equal distribution was observed for *SLC22A17* and *LCN2* among the tumors. Notably, *SLC22A17* expression showed the strongest association with favorable OS in SKCM patients (Log-rank test: 18.02), while *LCN2* displayed the strongest association with unfavorable OS in KIRC patients (Log-rank test: 12.77) (Figure 7A; Supplementary Table S9).

Interestingly, a significant and positive association (*p* ≤ 0.05) was found between *SLC22A17* expression levels and PFI in patients with GBM, LGG, LUAD, PAAD, PRAD, SKCM, and THYM, while ACC and READ PFI showed a negative correlation. An opposite trend was observed for *MMP9*, which decreased the PFI in four tumors, including ACC, GBM, KIRC, and THCA. Only the PFI of DLBC patients showed a positive correlation with *MMP9* expression levels (Figure 7B). Similar to OS analysis, the volcano plot of PFI showed that *LCN2* was both a favorable and unfavorable prognostic factor in an equal number of tumors. Interestingly, *LCN2* expression had the strongest association with favorable PFI in PRAD patients (Log-rank test 13.36) (Figure 7B; Supplementary Table S9).

### Methylation profiling of *LCN2*, *SLC22A17*, and *MMP9* in TCGA tumor samples

The involvement of DNA methylation in the regulation of *LCN2*, *SLC22A17*, and *MMP9* genes was investigated by analyzing the methylation status of each gene. To this purpose, the UCSC Xena tool was used to retrieve methylation levels of *LCN2*, *SLC22A17*, and *MMP9* GC probesets for all TCGA PAN-Cancer samples. Notably, no methylation data were available for the probesets cg01871963 and cg14631896 of *LCN2* and cg24406668, cg23353432, cg04145601, and cg14581475 relative to *MMP9*. The position of each CG probeset within the relative genes is represented in Supplementary Figure S2.

**FIGURE 8:**
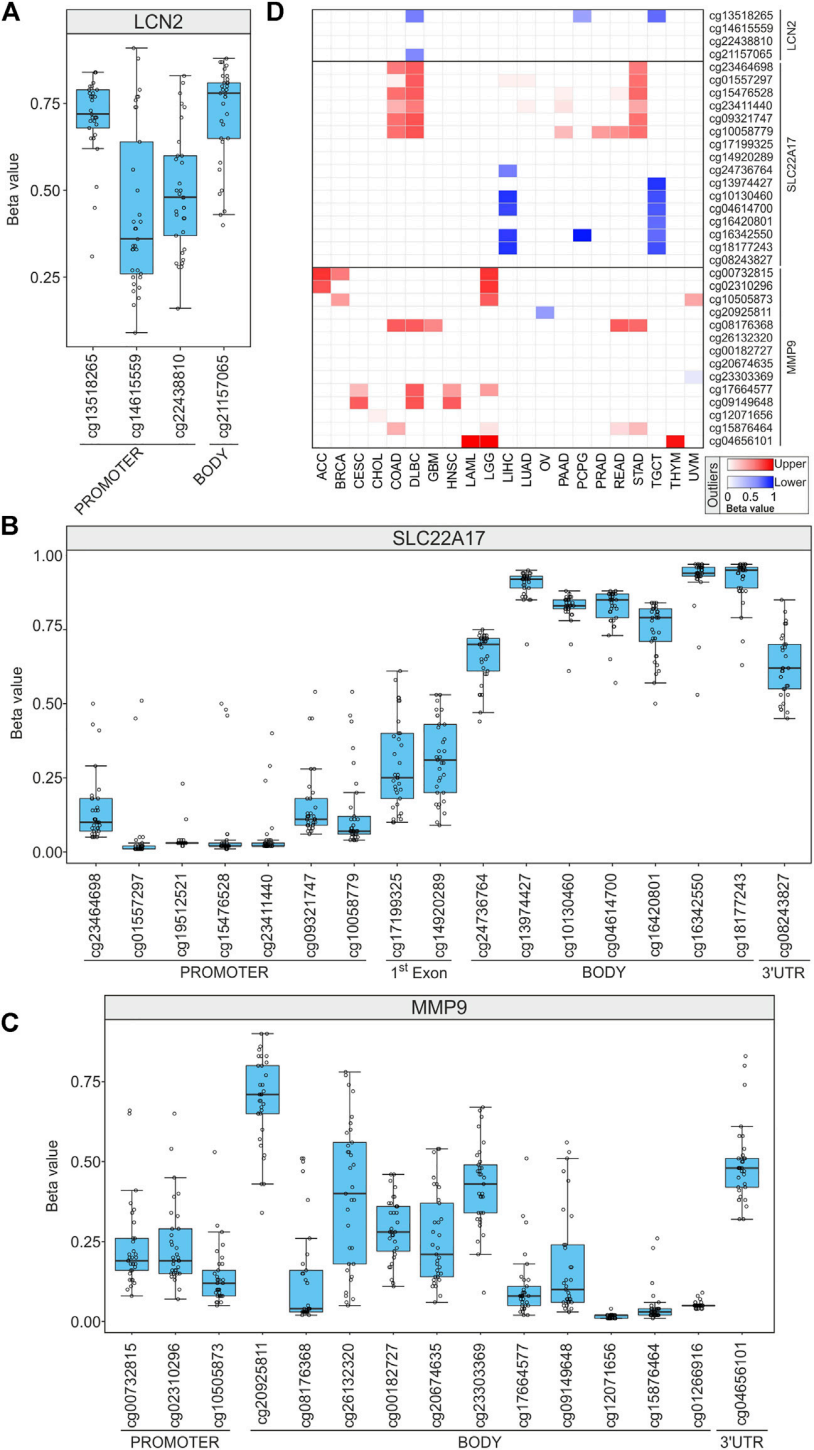
DNA methylation profiling of *LCN2*, *SLC22A17*, and *MMP9* in all tumor samples. **(A–C)** For each CG methylation probeset, the mean of methylation beta value is displayed for all tumor types (empty circles). **(D)** Heatmap showing the tumors in which the mean GC probeset beta value resulted lower (red) or upper (blue) outliers.

The Boxplot analysis revealed that *LCN2* CG probesets of the promoter region were methylated (median beta value ≥0.6) or partial methylated (0.6 < median beta value <0.2) in most TCGA tumor types, including the cg13518265 probeset that was highly methylated in 30 tumor types. Conversely, it was partially methylated in DLBC, PCPG, and TGCT also representing lower outliers in these tumor types (Figures 8A,D; Supplementary Table S10). In the *LCN2* body region, the cg21157065 probeset was largely methylated in 26 tumors and a lower outlier only in DLBC (Figures 8A and D; Supplementary Table S10).

The methylation profile analysis of *SLC22A17* revealed that the gene was widely hypomethylated (median beta values lower than 0.2) in the promoter region (from cg23464698 to cg10058779) in almost all tumors, except for COAD, DLBC, and STAD. Conversely, the 1st exon region showed partial methylation, and the body CG probesets (from cg24736764 to cg18177243) were highly methylated (beta value ≥0.6) (Figures 8B and D; Supplementary Table S10). Notably, almost all the body CG probesets showed lower methylation (below the 1st quartile) in LIHC and TGCT (Figure 8D). Finally, the 3′UTR region (cg08243827) was methylated in 19 tumors and partially in other 14 tumors (Figure 8B, Supplementary Table S10).

The methylation status of the *MMP9* promoter region (cg00732815, cg02310296, and cg10505873) was unmethylated (beta value ≤0.2) in more than 19 tumors. Conversely, both ACC and LGG were widely hypermethylated (upper outliers) (Figures 8C and D; Supplementary Table S10). Among the body CG probesets, the cg20925811 had the highest methylation levels in 26 tumors (beta value ≥ 0.6) such as ACC and LAML but was a lower outlier in OV (beta value: 0.34) (Figures 8C and D; Supplementary Table S10). Furthermore, some CG probesets of *MMP9* CpG63 island (cg26132320, cg00182727, cg20674635, and cg23303369) (Supplementary Figure S2) collectively showed moderate methylation levels in more than 50% of tumors. Finally, the 3′UTR region (cg04656101) was partially methylated in 29 tumors, while it was hypermethylated in LAML, LGG, and THYM (Figures 8C and D; Supplementary Table S10).

### Correlation analysis between DNA methylation and gene/isoform expression of *LCN2*, *SLC22A17*, and *MMP9* in TCGA tumor samples

**FIGURE 9:**
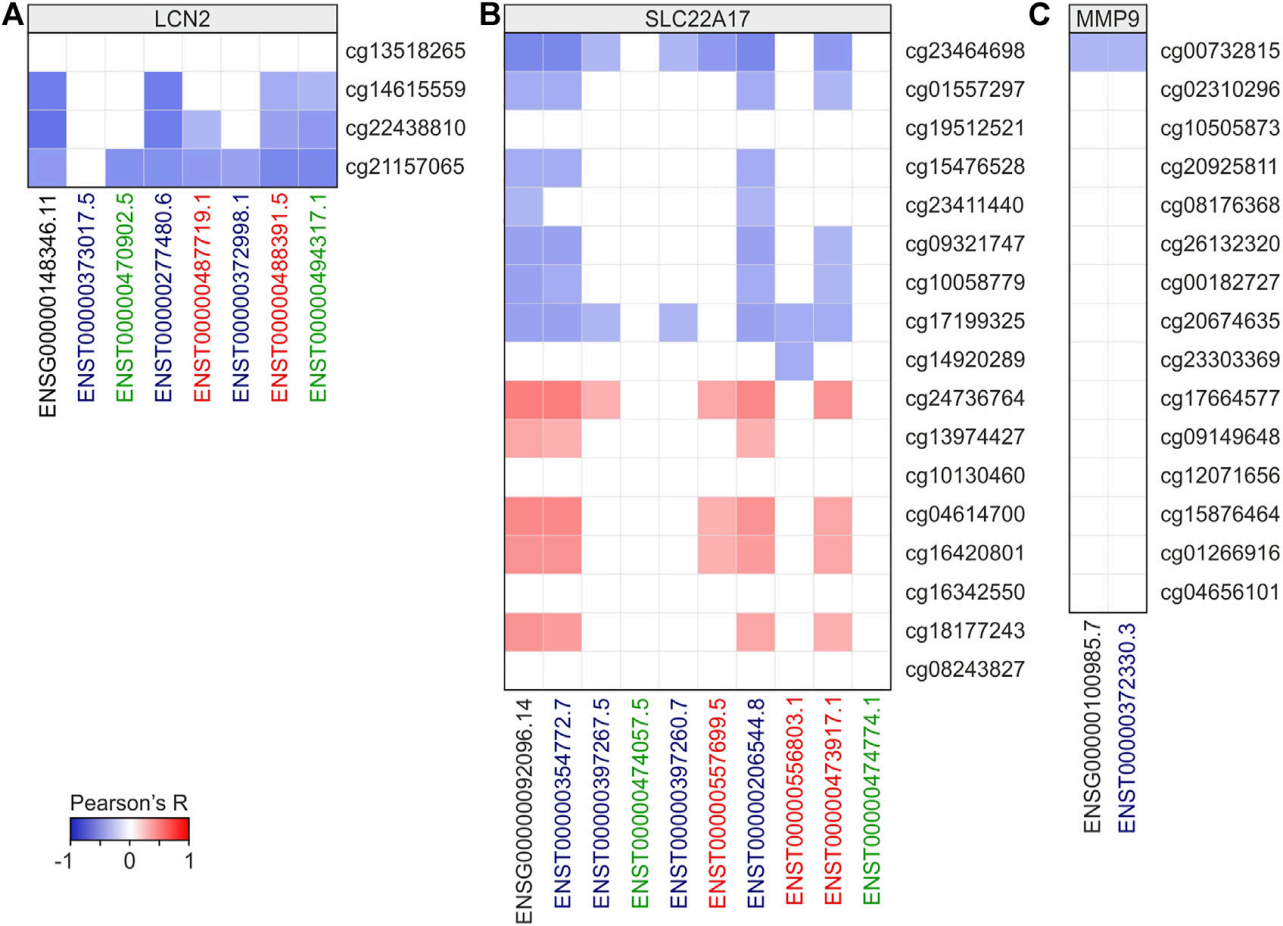
Correlation analysis between *LCN2* **(A)**, *SLC22A17* **(B)**, and *MMP9* **(C)** gene/isoforms expression and CG probesets methylation levels in all tumors. Correlation with Pearson’s r ≥ 0.3 or ≤ −0.3 and *p* ≤ 0.05 were included in the heatmap. Black bold labels indicate the *LCN2*, *SLC22A17*, and *MMP9* genes (ENSG). Coding ENST isoforms: blue; non-coding ENST isoforms: green; retained intron ENST isoforms: red.

To deeply investigate the role of DNA methylation in transcriptional regulation of *LCN2*, *SLC22A17*, and *MMP9*, correlation analysis was performed between CG probeset methylation levels and gene expression of *LCN2*, *SLC22A17*, and *MMP9* in all tumor samples (Figure 9; Supplementary Table S11). Furthermore, bearing in mind that methylation can affect both the activation of alternative transcription start sites and alternative splicing, correlation analysis was also extended to the expression of *LCN2*, *SLC22A17*, and *MMP9* isoforms (Figure 9; Supplementary Table S11). The correlation analysis for *LCN2* revealed that all CG probesets were negatively correlated (Pearson’s *r* ≤ −0.3; *p* ≤ 0.05) with gene and almost isoforms except for cg13518265. Conversely, no significant correlation was observed between all CG probesets and ENST00000373017.5 isoform (Figure 9A; Supplementary Table S11).

As regards *SC22A17*, the correlation analysis revealed an interesting correlation pattern identifying two different groups of CG probesets (promoter/1st exon and body/3′UTR regions, respectively) (Figure 9B). In particular, the first group was always negatively correlated to the expression of gene and some isoforms, including ENST00000354772.7, ENST00000206544.8, and ENST00000473917.1. At the same time, these isoforms were positively correlated (Pearson’s *r* ≥ 0.3 *p* ≤ 0.05) with the body/3′UTR CG probesets group (Figure 9B, Supplementary Table S11). As regards *MMP9*, only the cg00732815 probeset showed a negative correlation (Pearson’s *r* = −0.3) with *MMP9* expression (Figure 9C; Supplementary Table S11).

**FIGURE 10:**
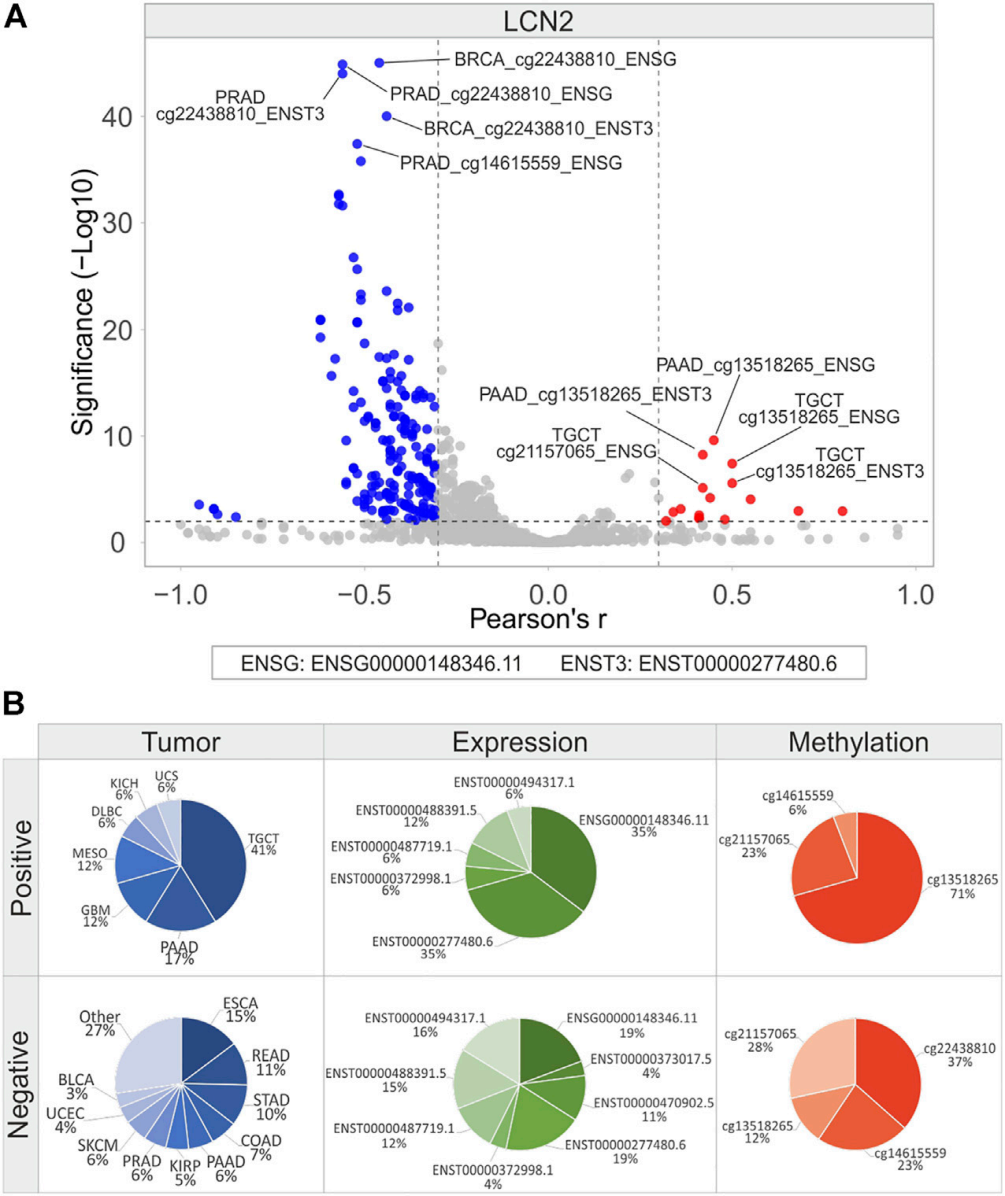
Correlation analysis between *LCN2* gene/isoforms expression and relative CG probesets methylation levels in each tumor type. **(A)** Volcano plot showing the correlation between CG probesets and gene/isoforms expression of *LCN2* (Pearson’s r ≥ 0.3 and ≤ −0.3, *p* ≤ 0.01). The top ten hits (five positive and five negative) based on correlation significance are labeled. Blue dots: negative correlation, red dots: positive correlation. **(B)** Percentage of tumor types, gene/isoforms and CG probesets showing Pearson’s r ≥ 0.3 and ≤ −0.3 (*p* ≤ 0.01), separately. No more than ten variables with the highest correlation values are indicated.

**FIGURE 11:**
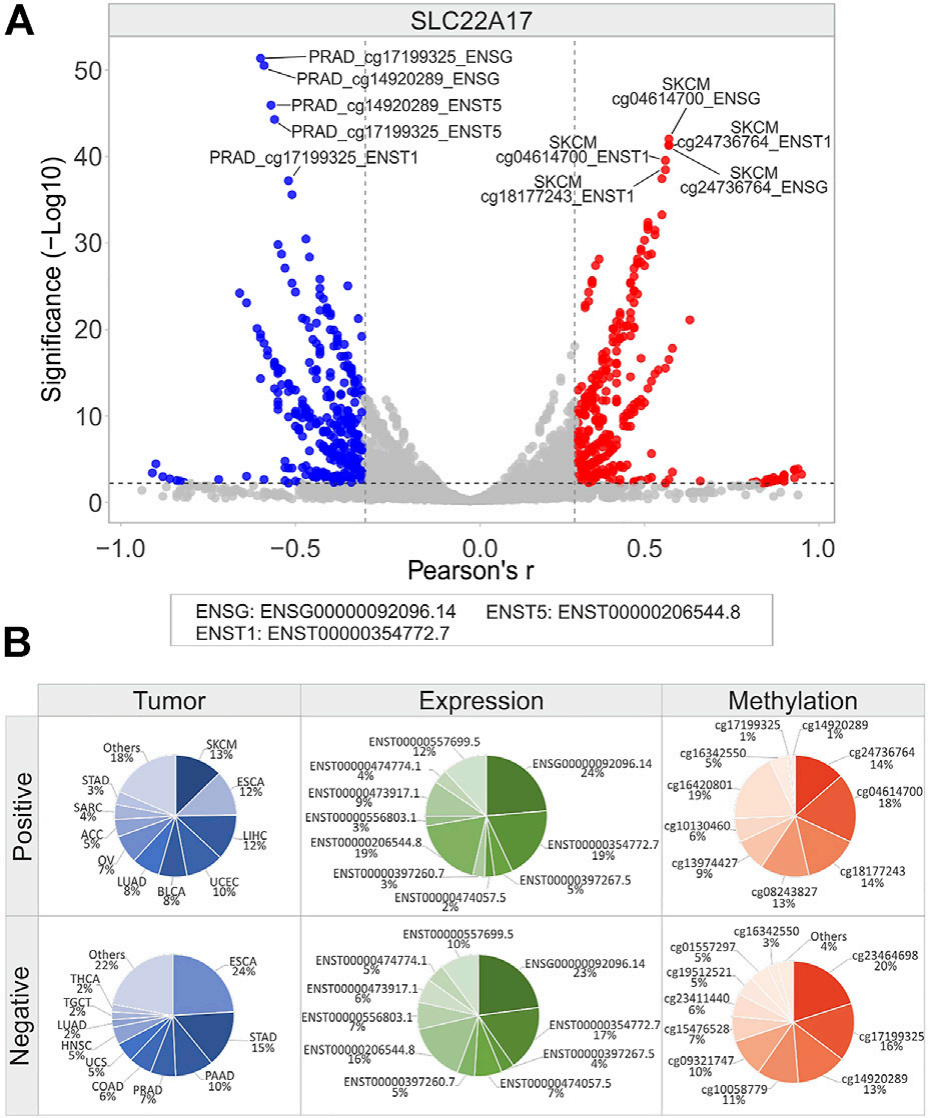
Correlation analysis between *SLC22A17* gene/isoforms expression and relative CG probesets methylation levels in each tumor type. **(A)** Volcano plot showing the correlation between CG probesets and gene/isoforms expression of *SLC22A17* (Pearson’s r ≥ 0.3 and ≤ −0.3, *p* ≤ 0.01). The top ten hits (five positive and five negative) based on correlation significance are labeled. Blue dots: negative correlation, red dots: positive correlation. **(B)** Percentage of tumor types, gene/isoforms and CG probesets showing Pearson’s r ≥ 0.3 and ≤ −0.3 (*p* ≤ 0.01), separately. No more than ten variables with the highest correlation values are indicated.

**FIGURE 12:**
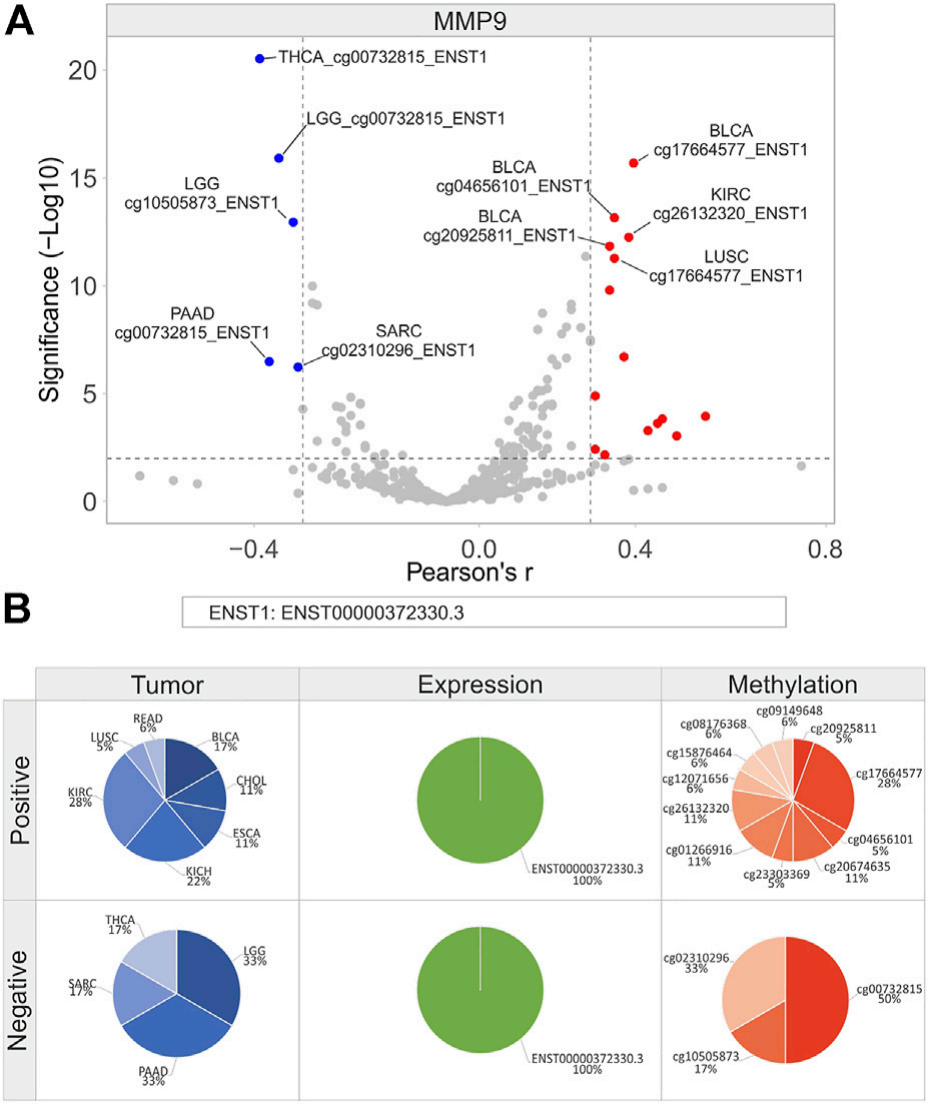
Correlation analysis between *MMP9* isoforms expression and relative CG probesets methylation levels in each tumor type. **(A)** Volcano plot showing the correlation between CG probesets and isoforms expression of *MMP9* (Pearson’s r ≥ 0.3 and ≤ −0.3, *p* ≤ 0.01). The top ten hits (five positive and five negative) based on correlation significance are labeled. Blue dots: negative correlation, red dots: positive correlation. **(B)** Percentage of tumor types, gene/isoforms and CG probesets showing Pearson’s r ≥ 0.3 and ≤ −0.3 (*p* ≤ 0.01), separately. No more than ten variables with the highest correlation values are indicated.

The correlation analysis was also carried out between the methylation levels and expression of *LCN2*, *SLC22A17*, and *MMP9* in each tumor type (Figures 10, 11, and 12; Supplementary Table S12). The Volcano plot analysis revealed that *LCN2* CG probesets mainly showed a negative correlation with *LCN2* gene/isoforms expression (Figure 10A). The highest number of negative correlation pairs was obtained for ESCA (15%) and READ (11%), for *LCN2* gene (19%) and ENST00000277480.6 (19%) isoform expression, and for the methylation levels of both cg22438810 (37%) and cg21157065 (28%) probesets. (Figure 10B). Among the positively correlated pairs, the *LNC2* gene and ENST00000277480.6 isoform expression were the most correlated along with the cg13518265 probeset (71%). Interestingly, the positive correlation pairs account for 41% in TGCT (Figure 10B).

Considering the correlation analysis for *SLC22A17*, it was observed an equal number of positive (*n* = 401) and negative (*n* = 419) correlation pairs (Figure 11A; Supplementary Table S12). Frequency analysis revealed that the tumor types showing the highest number of positive pairs were SKCM (13%), ESCA (12%), and LIHC (12%), while negative correlation pairs were more frequent in ESCA (24%) and STAD (15%). Interestingly, ENSG00000092096.14 and ENST00000354772.7 expressions were the most correlated in both positive and negative correlation pairs. Among *SLC22A17* CG probesets, the most represented in positive correlation pairs were all located in both body and 3′UTR regions, whereas those negatively correlated belonged to the promoter and 1st exon regions (Figure 11B).

As regards *MMP9*, a low number of correlation pairs was obtained while performing correlation analysis (Figure 12A). KIRC (28%) and KICH (22%) showed the highest number of positive correlation pairs, which included 10 of 11 CG probesets of the body region and 1 probeset of the 3′UTR region (Figure 12B). Among these, 3 CG probesets of the body region (CpG63 island: cg26132320, cg20674635, and cg23303369) showed a significant positive correlation in CHOL, KICH, and KIRC (Figure 12B; Supplementary Table S12). Conversely, all CG probesets in the promoter region were negatively correlated in LGG (33%), PAAD (33%), SARC (17%), and THCA (17%) (Figure 12B).

### Methylation pattern analysis of the *SLC22A17* promoter and body CG probesets in TCGA tumor types

**FIGURE 13:**
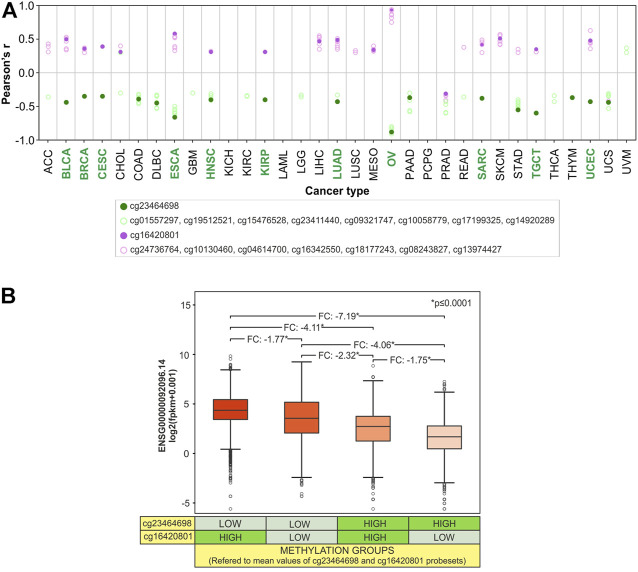
Synergic effect of promoter hypomethylation and body hypermethylation in the upregulation of *SLC22A17*. **(A)** Correlation analysis between gene expression (ENSG00000092096.14) and CG probesets methylation levels of *SLC22A17* was evaluated for each tumor type. Only CG probesets showing Pearson’s r ≥ 0.3 (magenta circles) and r ≤ −0.3 (green circles) *p* (≤0.05) are included. Magenta dots indicate the cg16420801 probeset while green dots represent the cg23464698 probeset. Green label indicates tumor types in which both CG probesets are significantly correlated. **(B)** Differential analysis (two-tailed unpaired *t*-test) of *SLC22A17* (ENSG00000092096.14) expression was performed stratifying the tumor samples in different four groups based on the methylation levels of cg16420801 and cg23464698 probesets with respect to the mean value calculated for each CG probeset in all tumor samples. FC and pValue are reported for each comparing group.

To evaluate if the methylation status of CG probesets within both promoter and body regions contributes to the regulation of *SLC22A17* expression, correlation analysis between CG probesets methylation and gene expression was performed for each tumor type (Figure 13A). Furthermore, to better understand how the *SLC22A17* expression was affected by the methylation levels of more correlated probesets, cg23464698 (promoter) and cg16420801 (body), the *SLC22A17* expression levels of all TCGA tumors were stratified in four methylation groups according to the methylation median values of both probesets (below and above median values) (Figure 13B).

The correlation analysis revealed that 15 tumors showed at least one CG probeset for each promoter/1st exon and body/3′UTR region correlated with *SLC22A17* expression (Figure 13A). As expected, the CG probesets of the promoter/1st exon region were mainly negatively correlated, whereas those of the body/3′UTR region were positively correlated in 14 tumors. Notably, the cg23464698 and cg16420801, the most frequent probesets correlated to *SLC22A17* expression in tumor types (Figure 11B), were simultaneously correlated in 11 tumors (Figure 13A, green labels). The stratification of tumor samples according to the methylation value of these probesets revealed that the expression levels of *SLC22A17* (ENSG) were the highest (median: 4.41, IQ range: 3.47–5.48) in Group 1 with hypomethylation of cg23464698 and hypermethylation of cg16420801 while the samples of Group 4 with the lowest *SLC22A17* expression levels (median: 1.72, IQ range: 0.51–2.82) showed an opposite trend of methylation levels for both CG probesets. Remarkably, a greater reduction in *SLC22A17* expression was observed in Group 3 than in Group 2, which was characterized by low cg23464698 (promoter) methylation levels (Figure 13B).

Overall, the results indicated that both promoter and body methylation are involved in the regulation of *SLC22A17* expression acting as transcriptional repressor and activator, respectively. In particular, a prominent role of DNA methylation in the promoter region can be inferred from this analysis.

### OS and PFI analyses associated with the methylation profiling of *LCN2*, *SLC22A17*, and *MMP9* in TCGA tumor samples

**FIGURE 14:**
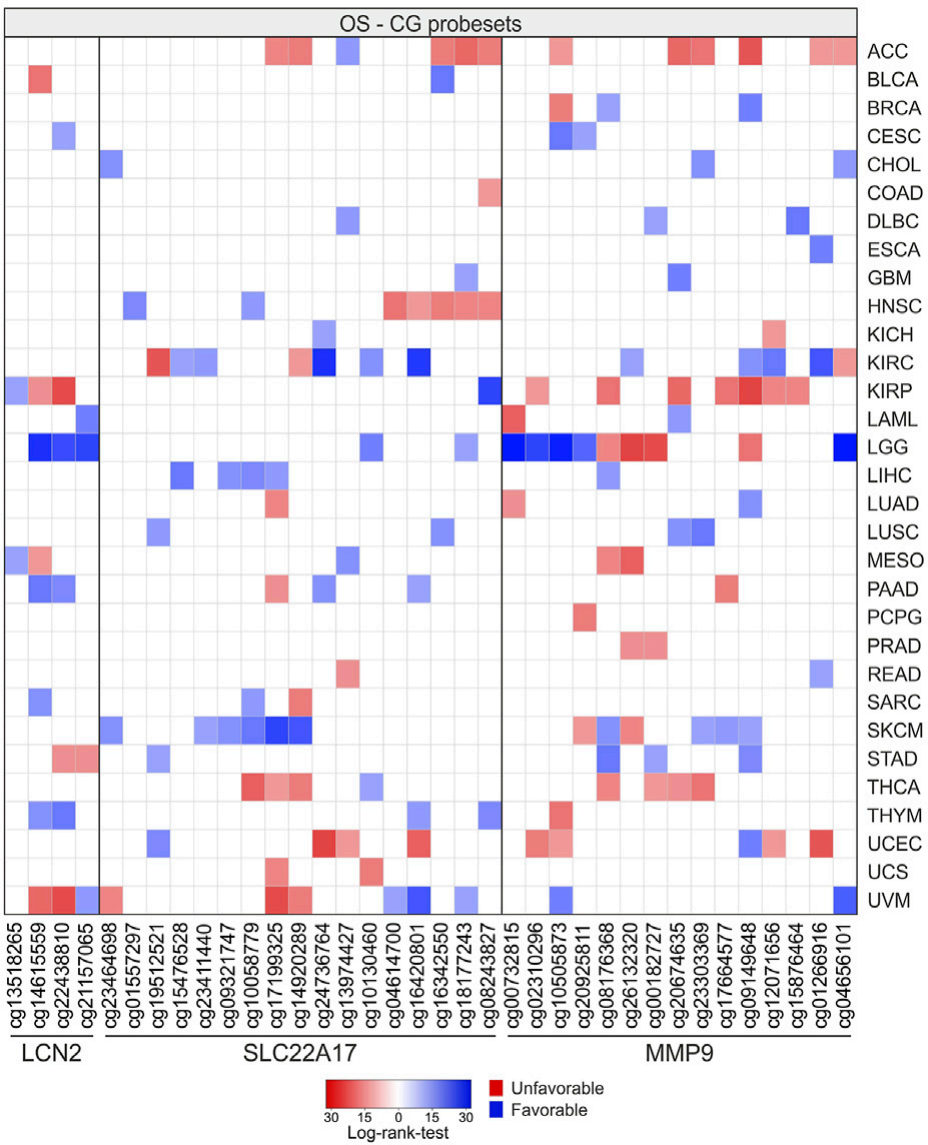
OS analysis according to methylation levels of CG probesets relative to *LCN2*, *SLC22A17*, and *MMP9* in all tumor types. The CG probesets statistically associated (*p* ≤ 0.05) with OS are represented in the heatmap.

Survival analysis was performed using Xena UCSC, retrieving the log-rank test values and p-values calculated for the CG probesets related to *LCN2*, *SLC22A17*, and *MMP9* in each tumor type (Figure 14; Supplementary Table S13). The analysis revealed that LGG, SKCM, KIRC, ACC, UVM, and KIRP displayed the highest number of CG probesets associated with OS. Among these, LGG and SKCM showed the highest number of CG probesets (*n* = 10) with favorable OS, whereas the CG probesets associated with poor prognosis were mainly represented in ACC (*n* = 11) and KIRP (*n* = 9) (Figure 14; Supplementary Table S13). In particular, three of four CG probesets of *LCN2* (cg14615559, cg22438810, and cg21157065) were favorable prognostic factors in LGG. As regards *SLC22A17* methylation status, a cluster of five CG probesets within the promoter and 1st exon regions were strongly associated with favorable OS in SKCM patients. Furthermore, the downstream CG probesets of *SLC22A17*, including the 3′UTR CG probeset, were associated with worse prognosis in HNSC (*n* = 5) and ACC (*n* = 3) (Figure 14; Supplementary Table S13). Regarding the OS analysis related to *MMP9* methylation status, the results revealed that about 50% of *MMP9* CG probesets were unfavorable factors in ACC and KIRP. An interesting trend was observed for LGG in which all promoter CG probesets and the first of the *MMP9* body region, as well as the 3′UTR CG probeset, were positively associated with OS, whereas the other body probesets (*n* = 4) were unfavorable factors (Figure 14; Supplementary Table S13).

**FIGURE 15:**
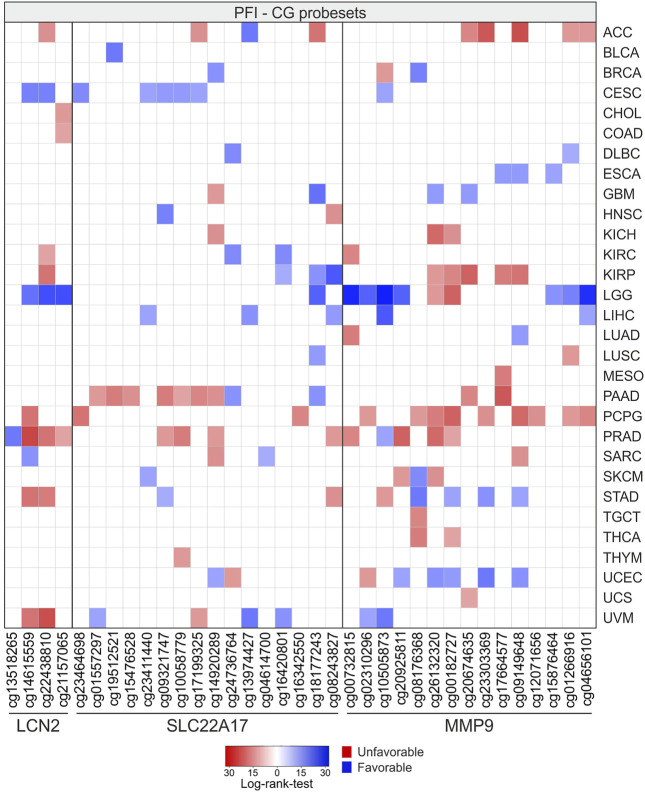
PFI analysis according to methylation levels of CG probesets relative to *LCN2*, *SLC22A17*, and *MMP9* in all tumor types. The CG probesets statistically associated (*p* ≤ 0.05) with PFI are represented in the heatmap.

Similar to OS analysis, the results of PFI analysis for LGG showed the highest number of CG probesets (*n* = 11) associated with a favorable PFI. Among these, three CG probesets were related to *LCN2*, whereas the others were mainly located in the promoter and downstream regions of *MMP9*. Conversely, PCPG showed the highest number of CG probesets (*n* = 12) significantly associated with PFI (*p* ≤ 0.05) with negative prognostic significance for *LCN2* (*n* = 1), *SLC22A17* (*n* = 2), and *MMP9* (*n* = 9) (Figure 15; Supplementary Table S14).

## Discussion

Cancer development and progression are closely influenced by TME, which regulates several tumor processes such as proliferation, invasiveness, metastasis, immune tolerance, and drug resistance (Whiteside, 2008). Many components of TME, including extracellular matrix, tumoral and surrounding cells, fibrillar proteins, and other soluble molecules, represent a source of biochemical and biomechanical stimuli that support tumor progression. In this field, the remodeling of TME with different approaches has become a research hotspot (Winkler et al., 2020). Over the past decades, it has been demonstrated that the *LCN2–SLC22A17–MMP9* pathway is implicated in TME modulation through the degradation of the adhesion proteins and several ECM components (Quintero-Fabián et al., 2019; Barillari, 2020). The activation of this pathway is also involved in the dysregulation of immunological synapses leading to the suppression of immune response (Juric et al., 2018). Furthermore, LCN2-SLC22A17 interaction could contribute to iron trafficking between TME and tumor cells, ensuring the iron supply and avoiding free iron accumulation, which may induce oxidative stress and ferroptosis (Chaudhary et al., 2021; Yao et al., 2021; Liu et al., 2021).

Interestingly, several studies reported that TME regulates the expression of *LCN2*, *SLC22A17*, and *MMP9*, which, in turn, modify the TME dynamism demonstrating a reciprocal modulation (Kessenbrock et al., 2010; Candido et al., 2016a; Wei et al., 2020; Crescenzi et al., 2021). This crosstalk is mediated by several chemokines, cytokines, and fibrillar proteins that activate the transcription of LCN2, SLC22A17, and MMP9 (Chinni et al., 2006; Iannetti et al., 2008; Xu et al., 2015). However, epigenetic alteration may contribute to enhancing or suppressing the signaling of these proteins (Lee et al., 2012; Abba et al., 2014; Falzone et al., 2016b; Cheng et al., 2020). In this context, DNA methylation represents the main regulation mechanism able to reprogram the expression pattern of key genes involved in carcinogenic processes. Several studies have demonstrated that DNA methylation is involved in aberrant expression of the *LCN2*, *SLC22A17*, and *MMP9* genes in various tumors (Cui et al., 2008; Falzone et al., 2016a; Zhang et al., 2021). However, the DNA methylation regulation of *LCN2* and *SLC22A17* has not been sufficiently investigated in cancer. To the best of our knowledge, multiscale integrated analysis based on the expression and DNA methylation of these genes has not been performed yet. This *in silico* approach could allow us to identify the role of the *LCN2–SLC22A17–MMP9* network in TME and its regulation mechanisms, including signaling pathway activation and epigenetic changes.

On these bases, the role of *LCN2*, *SLC22A17*, and *MMP9* was evaluated in different tumor types performing *in silico* analysis using the expression and methylation data available on TCGA and GTEx datasets. Furthermore, correlation and signaling pathway analyses were performed, including the protein expression values retrieved from the RPPA protein dataset.

The results of this study suggested that *LCN2*, *SLC22A17*, and *MMP9* genes interact with each other, indicating their synergic involvement in cancer. Interestingly, the expression of both *LNC2* and *MMP9* genes was increased in 16 tumor types, demonstrating their involvement in tumor development. This result was consistent with those reported in the literature for several tumors, including BLCA (Ricci et al., 2015; Candido et al., 2016b), CESC (Li et al., 2012), CHOL (Leelawat et al., 2011; Nair et al., 2018; Li et al., 2019), ESCA (Zhang et al., 2007), HNSC (Ruokolainen et al., 2005; Hiromoto et al., 2011; Stanciu et al., 2016; Verma et al., 2017), KIRP (Cho et al., 2003; Zhang et al., 2015), LIHC (Chen et al., 2012; Abdelsameea et al., 2020), LUAD (Ruiz-Morales et al., 2015), OV (Cho and Kim, 2009; Gupta et al., 2022); PAAD (Kaur et al., 2013), STAD (Du et al., 2011), UCEC (Cymbaluk-Płoska et al., 2017), COAD (Yang et al., 2014; Ozemir et al., 2016; Liang and Chang, 2018), LUSC (Lee et al., 2015), PRAD (Ma et al., 2017; Muşlu et al., 2017; Ulusoy et al., 2021), READ (Zhang et al., 2009; Annaházi et al., 2016), and THCA (Barresi et al., 2012; Liu et al., 2019; Xu et al., 2019).

Despite many authors reporting that *LNC2* and *MMP9* were widely associated with worse prognosis in several tumors, our *in silico* analysis revealed that the OS and PFI were simultaneously negatively affected by both genes only in KIRC patients.

The overexpression of *LCN2* and *MMP9* was consistently associated with the early promoter methylation status of both genes that have been found to be partially methylated in most tumor types. This evidence was corroborated by the opposite trend observed in the LGG tumor showing downregulation of *LCN2* and *MMP9* along with hypermethylation of the promoter region. Notably, *MMP9* overexpression was also associated with hypermethylation of CpG islands within the *MMP9* body region. These results were consistent with previous findings indicating a positive correlation between gene expression and intragenic DNA methylation of some genes dysregulated in melanoma, including *MMP9* (Candido et al., 2019), whose overexpression was significantly correlated with hypermethylation of the intragenic region (Falzone et al., 2016a; Klassen et al., 2018).

Unlike what has been observed for *LCN2* and *MMP9*, the *SLC22A17* expression was downregulated in most tumor types (66.7%) compared to normal tissue samples. However, the median expression of *SLC22A17* appeared moderately high in most tumors (Figure 1B, Supplementary Table S2). Of note, the expression of *SLC22A17* was increased in the neuronal tumors LGG, GBM, and PCPG and in THCA, which also showed high expression of *LCN2* and *MMP9*. The downregulation of *SLC22A17* observed in some tumors could be related to cancer survival and proliferation by affecting the cancer iron metabolism. For instance, the decrease of *SLC22A17* inhibits iron efflux from cancer cells resulting in iron-dependent proliferation (Hvidberg et al., 2005; Iannetti et al., 2008) and resistance to apoptosis (Devireddy et al., 2005). On the other hand, the intracellular iron overload induced by *SLC22A17* downregulation could have detrimental effects on cancer cell survival increasing oxidative stress and activating ferroptosis during pharmacological treatment (Lin et al., 2020; Wu et al., 2020). Notably, increased expression of *SL22A17* in GBM conferred increased sensitivity to treatment with temozolomide in combination with erastin, both of which activate ferroptosis through complementary mechanisms (Chen et al., 2015). Similarly, the *SLC22A17* expression could be predictive of response to ferroptosis drugs in both LGG and PCPG, which share the same embryonic cell origin as GBM (Liu et al., 2020; Zhao et al., 2021). Furthermore, the induction of ferroptosis in THCA, also overexpressing *SLC22A17*, may represent a potential therapeutic option (Wang et al., 2021). However, further studies are needed to verify if the *SLC22A17* overexpression confers resistance to ferroptosis in these tumors.

Based on this evidence, the blockage of *SLC22A17* could sensitize the cancer cells to iron-dependent death.

The expression pattern of *SLC22A17* appeared to be strongly associated with the DNA methylation status of this gene, suggesting that DNA methylation of the promoter strongly regulates gene expression. Indeed, the hypomethylation of the *SLC22A17* promoter largely observed in tumor samples supported the moderately high *SLC22A17* expression found in most tumor types. These results were further confirmed by the increased methylation levels of *SLC22A17* promoter, which was associated with low expression levels of *SLC22A17* in DLBC, STAD, and COAD. Interestingly, our results indicate that intragenic hypermethylation synergically contributes to *SLC22A17* gene regulation along with promoter methylation (Figure 13).

The correlation analysis performed on all TCGA tumor types highlighted a negative correlation between *LCN2* and *SLC22A17* expression. In particular, the gastrointestinal tumors (ESCA, STAD, READ, and COAD) showed both the highest expression of *LCN2* and the lowest expression of *SLC22A17* compared to the related normal tissues. Notably, the expression levels of *MMP9* were also strongly upregulated in these tumors, suggesting a key role of the LCN2–SLC22A17–MMP9 network in gastrointestinal tumors. This expression pattern was significantly correlated with both FN1 and CLDN7 RPPA protein levels in READ, indicating that the LCN2–SLC22A17–MMP9 network is involved in the TME regulation of this tumor. FN1 and CLDN7 have been investigated as TME factors involved in the epithelial to mesenchymal transition (EMT) and metastasis in different tumor types (Li et al., 2017; Wang et al., 2019). As observed in protein network analysis, both proteins interact with CDH1, a hallmark of EMT in cancers (Lioni et al., 2007). Therefore, it can be speculated that FN1 and CLDN7 proteins may mediate tumor progression through the network interaction with LCN2 and MMP9 (Sen et al., 2010; Pope et al., 2014; Chung et al., 2016). In this context, LAML, DLBC, SKCM, TGCT, and THYM tumors displayed a different trend in which *LCN2* also decreased along with *SLC22A17*. Notably, the decrease in *LCN2* expression was associated with the hypermethylation of *LCN2* promoter in these tumors.

Of note, *LCN2*, *SLC22A17*, and *MMP9* genes were found to be markedly upregulated in THCA, which also showed hypomethylation in the promoter region of all genes. Among these genes, only *MMP9* was significantly correlated with worse PFI outcome in THCA patients. These data are in agreement with what has been reported in the literature (Liu et al., 2019; Xu et al., 2019).

To the best of our knowledge, this study is the first that explored the epigenetic mechanisms of *LCN2*, *SLC22A17*, and *MMP9* regulation at the same time and in all TCGA tumors. The results obtained from these *in silico* analyses allowed us to identify a common trend of some genetic and epigenetic factors able to alter the *LCN2–SLC22A17–MMP9* network within the TME of different tumor types. Specifically, our findings demonstrated that the expression of *LCN2* and *MMP9* was associated with the development of main tumor types. Conversely, the *SLC22A17* expression was mainly downregulated in tumor samples showing different regulation mechanisms compared to *LCN2* and *MMP9*. Interestingly, the overall expression profile of these genes in tumors was often system- or organ-specific and dependent on epigenetic mechanisms, thus, identifying specific DNA methylation patterns in TCGA tumors. However, the final comprehensive interpretation of results cannot be fully achieved for all tumor types simultaneously due to the great variability observed among tumors.

This study provides an overview on the epigenetic regulation of *LCN2*, *SLC22A17*, and *MMP9* in the major tumor types allowing the identification of novel DNA methylation biomarkers and potential epi-drug targets of TME involved in tumor progression and drug resistance.

## Data Availability

The datasets presented in this study can be found in online repositories. The names of the repository/repositories and accession number(s) can be found in the article/Supplementary Material.
