# Supplementary figures and images for: Bioinformatic analysis of the LCN2–SLC22A17–MMP9 network in cancer: The role of DNA methylation in the modulation of tumor microenvironment
Source: Front Cell Dev Biol. 2022 Sep 21;10:945586. doi: 10.3389/fcell.2022.945586 (PMC9532607; doi:10.3389/fcell.2022.945586)

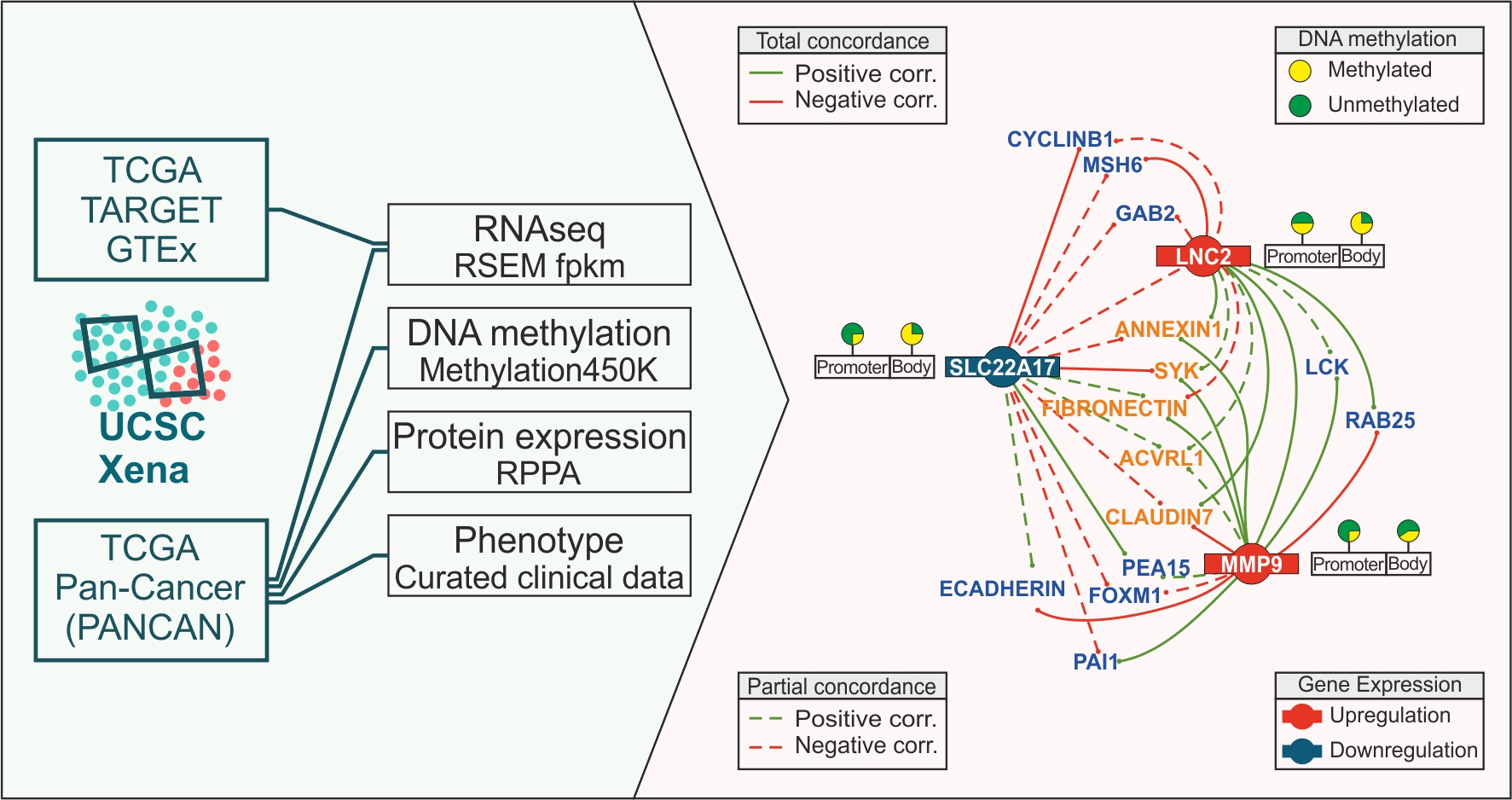

Supplement: Supplementary file 1 [file Image1.JPEG]
