# Supplementary material for: Bioinformatic analysis of the LCN2–SLC22A17–MMP9 network in cancer: The role of DNA methylation in the modulation of tumor microenvironment
Source: Front Cell Dev Biol. 2022 Sep 21;10:945586. doi: 10.3389/fcell.2022.945586 (PMC9532607; doi:10.3389/fcell.2022.945586)
Supplement: Supplementary file 3 [file DataSheet1.DOCX]

Supplementary Material

# Supplementary Figures

**
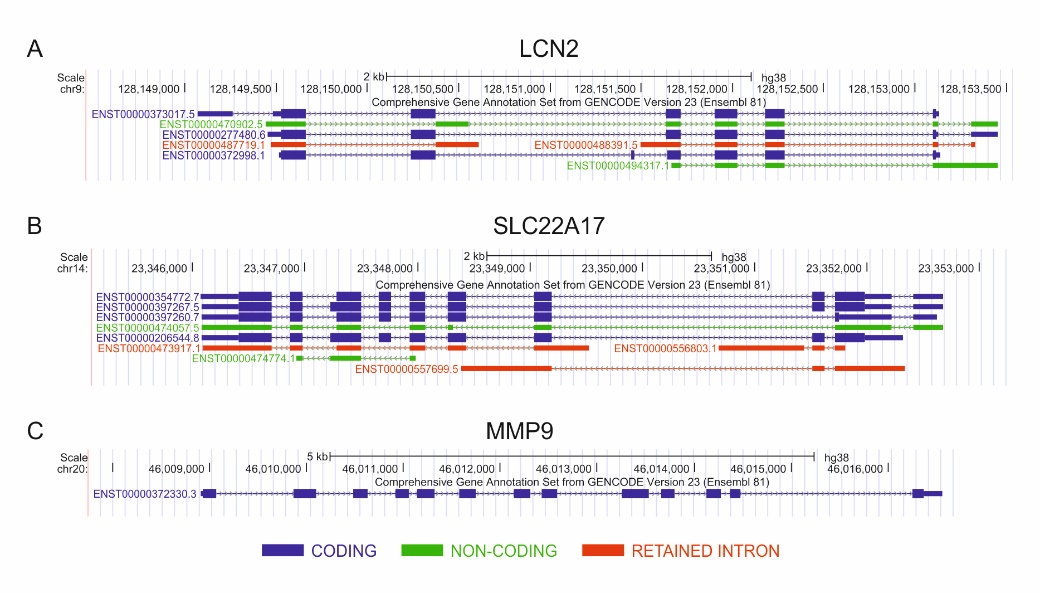
**

**Figure S1. Annotation of LCN2, SLC22A17, and MMP9 isoforms.** UCSC tool was used to retrieve the GENECODE annotations (version 23- Ensembl 81). Coding isoforms: blue; non-coding isoforms: green; retained intron isoforms: red.


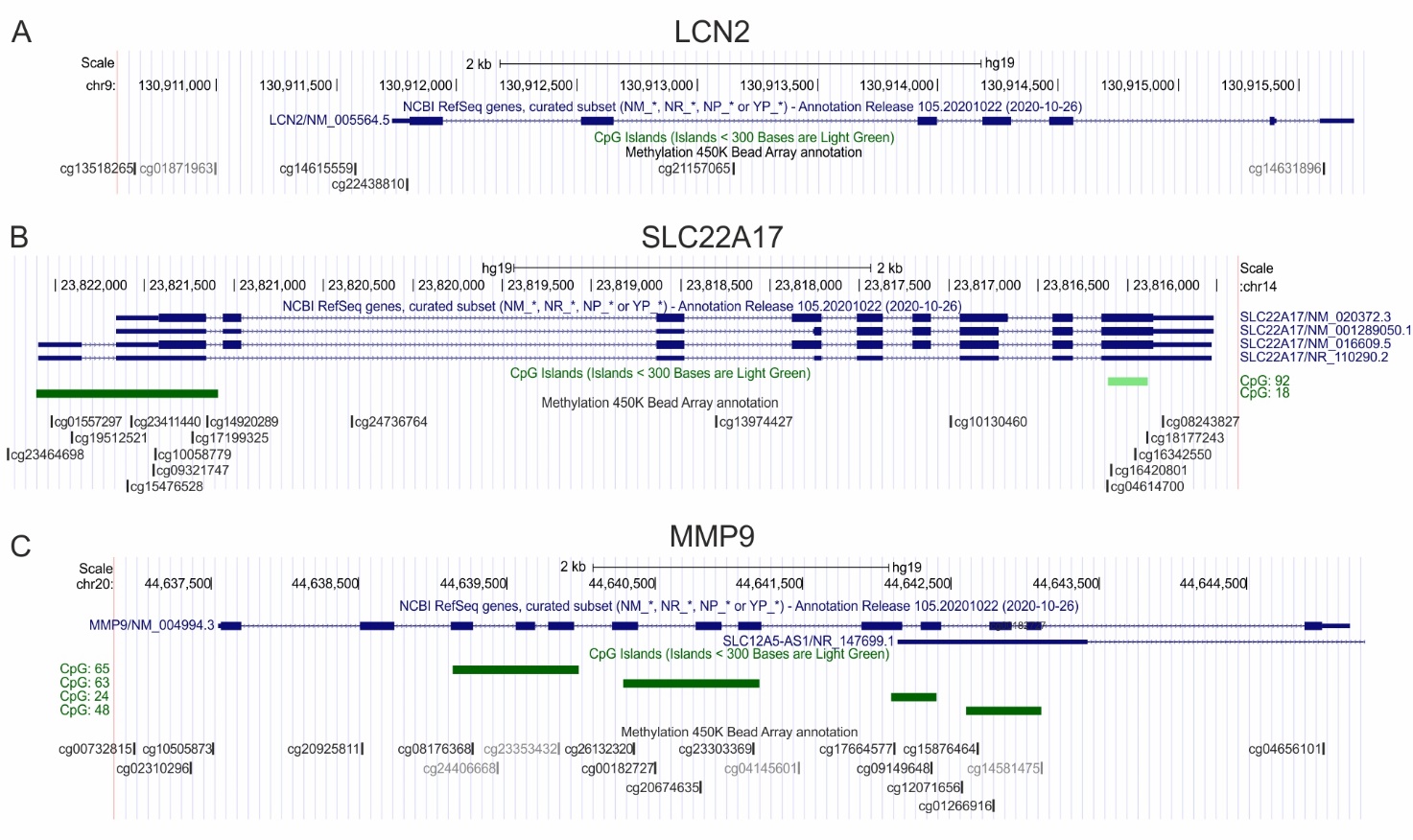


**Figure S2. UCSC visualization of LCN2, SLC22A17, and MMP9 CG probesets.** The light gray CG probesets were not available from TCGA Pan-Cancer dataset. CpG islands were also indicate in green according to UCSC setting.

# List of supplementary tables (excel file):

Table S1. List of tumor types and normal tissues included in TCGA Pan-Cancer and TCGA TARGET GTEx cohorts

Table S2. Median levels of gene and isoforms expression for each tumor types (TCGA) and matched normal tissues (GTEx)

Table S3. Differential analysis of LCN2, SLC22A17, and MMP9 genes and isoforms expression between tumors (TCGA) and matched normal tissues (GTEx)

Table S4. Correlation analysis between LCN2, SLC22A17, and MMP9 genes and isoforms expression in tumor (TCGA) and normal (GTEx) samples

Table S5. Correlation analysis between LCN2, SLC22A17, and MMP9 genes and isoforms expression and protein levels in tumor (TCGA) samples

Table S6. List of RPPA proteins included in correlation analysis. The proteins are divided into 9 Groups according to the tumor types in which were analyzed

Table S7. Kegg pathway enrichment analysis of genes and informs selected by average linkage clustering analysis in all tumor samples

Table S8. Correlation analysis between LCN2, SLC22A17, and MMP9 gene expresssion and protein levels by cancer type

Table S9. Overall Survival (OS) and Progression Free Interval (PFI) analyses for each tumor type according to the LCN2, SCL22A17, and MMP9 gene expression

Table S10. Median of CG probesets beta value of LCN2, SLC22A17, and MMP9 calulated for each tumor type

Table S11. Correlation analysis between gene/isoforms and CG probesets of LCN2, SLC22A17, and MMP9 in all tumor samples

Table S12. Correlation analysis between gene/isoforms and CG probesets of LCN2, SLC22A17, and MMP9 in all tumor types

Table S13. OS analysis according to CG probesets of LCN2, SLC22A17, and MMP9 in all tumor types

Table S14. PFI analysis according to CG probesets of LCN2, SLC22A17, and MMP9 in all tumor types
